# Integrated network model provides new insights into castration-resistant prostate cancer

**DOI:** 10.1038/srep17280

**Published:** 2015-11-25

**Authors:** Yanling Hu, Yinmin Gu, Huimin Wang, Yuanjie Huang, Yi Ming Zou

**Affiliations:** 1 Experimental Centre of Medical Sciences, Guangxi Medical University, Nanning, Guangxi, 530021, China; 2 Center for Genomic and Personalized Medicine, Guangxi Medical University, Nanning, Guangxi, 530021, China; 3 Department of Mathematical Sciences, University of Wisconsin-Milwaukee, Milwaukee, WI, 53201, USA

## Abstract

Castration-resistant prostate cancer (CRPC) is the main challenge for prostate cancer treatment. Recent studies have indicated that extending the treatments to simultaneously targeting different pathways could provide better approaches. To better understand the regulatory functions of different pathways, a system-wide study of CRPC regulation is necessary. For this purpose, we constructed a comprehensive CRPC regulatory network by integrating multiple pathways such as the MEK/ERK and the PI3K/AKT pathways. We studied the feedback loops of this network and found that AKT was involved in all detected negative feedback loops. We translated the network into a predictive Boolean model and analyzed the stable states and the control effects of genes using novel methods. We found that the stable states naturally divide into two obvious groups characterizing PC3 and DU145 cells respectively. Stable state analysis further revealed that several critical genes, such as PTEN, AKT, RAF, and CDKN2A, had distinct expression behaviors in different clusters. Our model predicted the control effects of many genes. We used several public datasets as well as FHL2 overexpression to verify our finding. The results of this study can help in identifying potential therapeutic targets, especially simultaneous targets of multiple pathways, for CRPC.

Prostate cancer (PCa) is one of the most commonly diagnosed lethal cancers and the leading cause of cancer death for men worldwide. Reducing testosterone concentration is a common treatment for advanced PCa^1^. However, the cancer usually recurs and gradually becomes castration-resistant prostate cancer (CRPC) under this treatment. A better understanding of the regulation of CRPC would improve prognosis in prostate cancer^2^,^3^.

Recent studies^1^,^4^,^5^ have suggested that cancer is not only a disease with a genetic basis, but is also driven by perturbations at the signaling network level. Therefore, developing treatments that target multiple pathways in CRPC regulation could potentially provide more effective approaches to treating CRPC^6^. However, though the biological mechanisms of PCa have been an intensively studied subject, experimental results were often focused on limited interactions in one or two pathways due to the fact that experiments are high-cost and time-consuming. In this study, in order to better understand the molecular mechanism of CRPC, we integrated the existing signaling pathway information to investigate CRPC gene regulation using a system-wide approach^7^.

There exists a promising approach for a system-wide study of a gene regulation system^6^,^7^,^8^. In this approach, one will first construct a comprehensive regulatory network utilizing existing information in the published literature, and then translate the network into a predictive Boolean model to perform further analysis and thus obtain information encoded in the network. In such a gene regulatory network, the proteins, the transcripts, and the small molecules in the regulatory pathways form the nodes of the network, and the interactions among them are indicated using directed edges. The analysis of the network provides insights, sometimes unexpected, to guide further experiments and drug developments^8^,^9^,^10^,^11^. While the topological properties of a gene regulatory network can be studied using algorithms in graph theory, Boolean models offer an effective approach for the study of the dynamical information of the network when it is considered as a discrete dynamical system. Due to the fact that almost all (if not all) published literature in CRPC related regulation studies provides only “suppress” or “induce” information on gene interactions, Boolean models, in which each node assumes “ON” or “OFF” states, are suitable choices for the modeling of CRPC regulation system.

Adopting the approach described above, we constructed a comprehensive CRPC regulatory network and studied its dynamical properties using a novel approach, which combines the detection and statistical analysis of all stable states of a Boolean model of the network. We also applied a new efficient computational method to investigate the control effects of the genes using the Boolean model.

## Results

### The CRPC regulatory network

**Figure 1:**
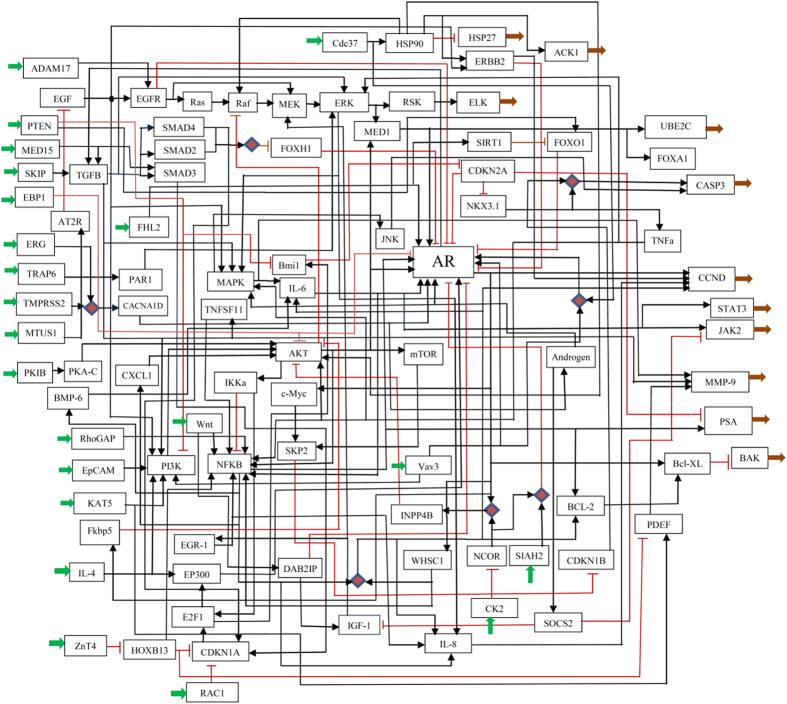
The constructed CRPC signaling network. The squares (nodes) represent the genes or proteins with their abbreviations. A thick green arrow indicates an input, a thick brown arrow indicates an output, a black line indicates an activation, a red blunt-ended line indicates an inhibition, and a red diamond indicates an AND connection which all involved nodes need to present to trigger the downstream event. Full names of the genes or proteins are given in Table S2 and their expression levels are given in Table S3.

We performed a literature search using PubMed with the search terms: “androgen resistant”, “androgen independent”, “AR independent”, “AR resistant”, “castration-resistant”, “PC3”, “DU145”, and “prostate cancer”, which delivered 5,115 abstracts. We selected 246 pairs of gene-gene, gene-protein, and protein-protein interactions and the corresponding genes and proteins from 119 references. The selection was based on whether the information on “promotes” (or “activates” or “induces” or “stimulates” or “responses” or “recruits” or “enriches” or “inhibits”) or “suppresses” (or “degrades” or “blocks”) was conclusive in the reference(s). References that provide only information such as “increases” were not included. The selected genes and their interactions are summarized in Table S1, in which the source nodes, the target nodes, two qualifiers of relationships and the corresponding references are listed. The full names of the nodes as well as the abbreviations are given in Table S2. However, this procedure resulted in a disconnected network as well as some simple connections that clearly do not offer integrated information for the whole system. Since further search of the literature did not lead to extra information to help incorporating these small components and simple connections into the main network, they were ignored in our final construction of the network, which consists of 91 nodes and 182 interactions (Fig. 1). Among these 91 nodes, many have their expression level information available in the literature. These are summarized in Table S3. We used the information in Table S3 to provide additional verification for the predictive ability of our Boolean model.

### Topological properties of the CRPC network

We analyzed the topological properties of our CRPC network with the help of CellNetAnalyzer^12^. The analysis detected a strongly connected component, i.e. a subset of nodes in which every node can be reached from any other node in the subset. This strongly connected component consists of 30 nodes (TGFB, SMAD2, SMAD3, SMAD4, Raf, MEK, ERK, FOXH1, MED1, FOXO1, CDKN2A, NKX3.1, TNFa, MAPK, TNFSF11, IL-6, Bmil, IL-6, AR, BMP-6, CXCL1, IKKa, PI3K, NFKB, Fkbp5, EP300, E2F1, CDKN1A, INPP4B, and WHSC1). The analysis also detected 181 feedback loops, which include 62 negative feedback loops. The 30 nodes that form the strongly connected component all participate in the feedback loops (Fig. S1). We observed that SMAD2 and FOXH1 participate only in the positive feedback loops, AR participates in 95% of the feedback loops and in 97% of the negative ones. We summarize the main finding of our analysis results as follows (see Table S4 for details): (i) Kinases MEK and ERK1/2 down-regulate their own activation through the phosphorylation of MAPK. (ii) The phosphorylation of MAPK incurs IL-6 expression, and promotes AR production. (iii) AKT positively influences AR via the activation of IKKa-E2F1-BMI1 cascade. (IV) AKT influences IKKa’s phosphorylation and further affects NF-κB nuclear translocation, and thus promotes AR production. (V) AKT presents in all detected negative feedback loops. (VI) The genes AR, ERK, TGFR, IL-6, AKT, NFKB, and E2F1, participate the most in the feedback loops (Fig. S1). Among these, ERK, IL-6, AKT, NFKB, and E2F1 up-regulate AR. AR up-regulates TGFB, which in turn down-regulates FOXH1, an inhibitor of AR^13^,^14^,^15^,^16^,^17^,^18^,^19^.

### Analysis of the stable states via clustering

**Figure 2:**
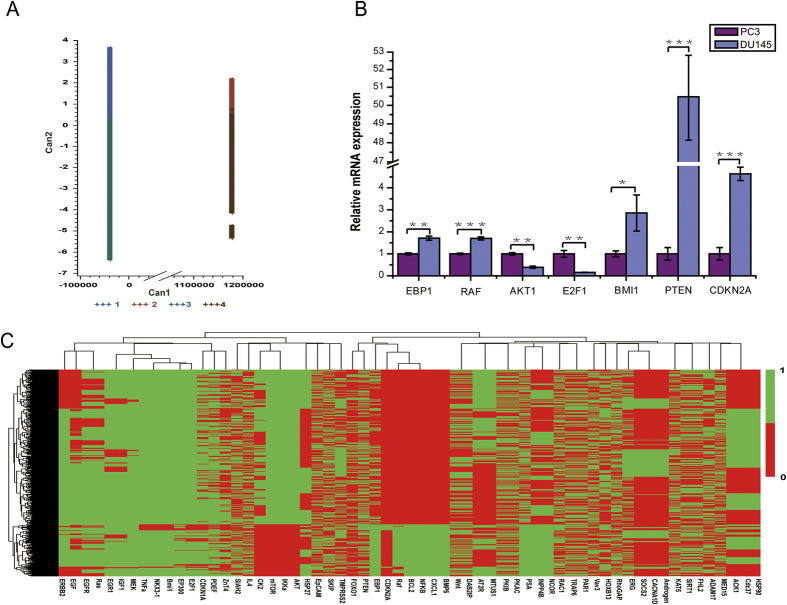
Clusters of the stable states and experimental result. (**A**) The clusters were obtained by using SAS Proc Fastclus. CAN1 denotes the linear combination of all variables that provides the greatest difference (in terms of a univariate F test) between the class means, and Can2 provides the greatest difference between class means while being uncorrelated with Can1. The symbol +++1 indicates the color scale for group 1; similarly for the other three groups. (**B**) Heatmap cluster for 12,254 stable states over 12 genes. Rows denote stable states and columns indicate gene expression states. Each gene takes only two states: “on” (green) or “off” (red). (**C**) qRT-PCR showed that the same genes behaved differently in PC3 and DU145 respectively. The asterisks specify the p value ranges: *indicates 0.01 < p value < 0.1,**indicates 0.001 < p value < 0.01, ***indicates p value < 0.001. The error bars depict the standard error of the mean of three replicates.

A Boolean model’s dynamics can be studied by analyzing its stable states^7^,^8^,^10^,^12^,^20^. Crespo *et al.*^21^ have proposed a method to predict expression values of genes involved in stable cellular phenotypes in a literature-based gene regulatory network while maintaining the consistency between the predicted stable states and the known stable states. Usually, a Boolean model of a literature-based gene regulatory network implies a large number of stable states, while in reality, there are only a few distinct states of a living organism that can be observed. Therefore, instead of treating each stable state of a Boolean model of a biological system as a possible outcome, we proposed to classify them using suitable cluster methods and treat each of the clusters as a possible single outcome. We did the cluster analysis using SAS in three steps: First, we used *proc standard* to standardize all 91 variable to a mean of 0 and standard deviation of 1. Second, we used *proc fastclus* to create a set which we named Clust and used Euclidean distance to run the clustering. Third, we used the CANDISC and GPLOT procedures to generate a graph presentation for the clustering result. This clustering method uses a nearest centroid sorting algorithm inspired by Hartigan’s (1975) leader algorithm and MacQueen’s (1967) k-means algorithm. Our computation detected 33,554,432 stable states and we found that they were clustered into two distinct groups. The clustered result showed in Fig. 2A consists of 4 clusters of sizes 22,070,480, 814,568, 10,435,376, and 234,008, respectively. These 4 clusters are naturally divided into two distinct groups: clusters 1 and 3 were in one group and clusters 2 and 4 were in the other. Guided by the clustering result, we performed a simple statistical analysis on these clusters by computing the rates of the expressions (0 or 1) of each node in the clusters and found that the rates of each node in clusters 2 and 4 (or in clusters 1 and 3) were similar and the rates were different between the two groups. We also computed the ratios of the rates of group 1 (clusters 2 and 4) over group 2 (clusters 1 and 3). Table 1 presents the results for the genes whose ratios were >1.2 or <0.8.

It was reported in the literature^22^ that no PTEN expression was detected in PC3 cells, which was correlated to the high levels of phosphorylated AKT; whereas high levels of PTEN were detected in DU145 cells, which was correlated to the fact that no detectable level of phosphorylated AKT in DU145. In our computed results, the rate of “1” of PTEN in group 1 was greater than the rate in group 2, and the ratio (of group 1 over group 2) was 2.07. The rate of “1” of AKT in group 2 was greater than the rate in group 1, and the ratio (of group 1 over group 2) was 0.55. This suggested that group 1 represented DU145 and group 2 represented PC3. In order to use heatmap clustering to analyze the stable states, we needed to reduce the number of stable states since we were unable to run heatmap clustering for all detected stable states. We found that we could run heatmap clustering efficiently for ~12,000 stable states. Thus, we randomly selected 12,254 stable states from the computed 33,554,428 stable states and ran clustering analysis for 61 genes that were not always “0” (or “1”) in all 33,554,428 stable states (the other genes’ expressions were always either 0 or 1). Our results showed that the ratios of the expressed rates of most genes of group 1 over group 2 (clusters 2–4/clusters 1–3) in Table 1 are consistent with the heatmap clustering result (Fig. 2C). This can be seen, for example, in the cases of Raf, AKT, NFKB, CXCL-1, and so on.

To verify our computed result and provide supporting evidence for our network and Boolean model, we searched genome-wide expression datasets from GEO (Gene Expression Omnibus, http://www.ncbi.nlm.nih.gov/geo/) using the search expression “PC3 and DU145”, and selected dataset GSE41445, which contains 3 repeats, to use for validation. We remark that this selected information was not used for the construction of our network. As usual, we call the ratio of gene expression values “fold-change” and call its logarithm “log fold-change” (logFC), and we use the signs “+” and “−” to indicate up or down expressions respectively. We used GEO2R, which is accessible at NCBI GEO website^23^, to compute logFC. If several probes corresponded to a gene, we selected the probe with the highest variation. The last two columns of Table 1 show that except for Bmi1, NKX3.1 and AKT/mTOR, the ratios are consistent (up to different scales) with our computed result. The inconsistency shown by AKT/mTOR could be due to the fact that the AKT/mTOR family includes several members, and there were several probes for each gene.

We took another step to validate our results. We used qRT-PCR to measure the following 7 genes on PC3 and DU145 Cells: EBP1, RAF, AKT1, E2F1, Bmi1, CDKN2A, and PTEN. The ratios of DU145 over PC3 were 1.74, 1.57, 0.41, 0.17, 3.00, 4.63, and 65.51 for these 7 genes respectively (see Fig. 2B). The measured result was also consistent with the computed result except that Bmi1 showed an inconsistency here too. We observed that in the constructed CRPC network, Bmi1 has only two upstream regulators: a promoter E2F1 and an inhibiter PTEN. Since the rate and ratio for E2F1 were low (here E2F1 had a ratio 0.17, the lowest among the 7 genes), the corresponding numbers in Bmi1 should also be low according to the network. This suggested that more experimental data would be needed to find out if there are more genes interacting with Bmi1 directly in the system, especially the ones that regulate Bmi1. However, a further literature search did not lead to extra up-stream genes for Bmi1. Aside from this, the evidence supported the simulation results.

### Control effects of the genes

**Figure 3:**
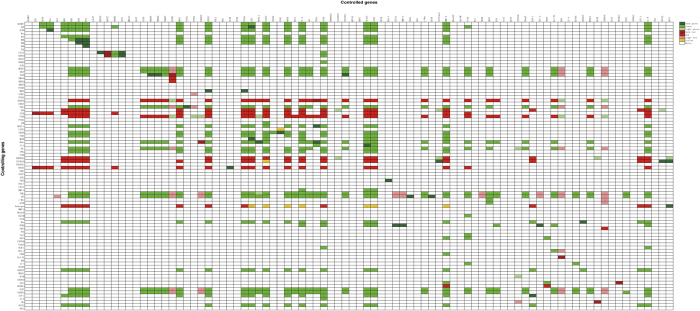
Control effects of the genes in the constructed CRPC regulatory network. “Controlling Genes” are labeled vertically; “Controlled Genes” are labeled horizontally. Each color scale is assigned a number for easy recognition. The effects on the Controlled Genes through the perturbation of each Controlling Gene are indicated by the corresponding row. The meanings of the colors of the matrix entries (*i*th row and *j*th column) are: Dark green box = 3, means node *j* is co-expressed with node *i*; Green box = 2, means when node *i* is “0”, node *j* is not a constant, and node *i* is “1” results in node *j* being “1”; Light green box = 1, means node *i* is “0” results in node *j* being “0”, and when node *i* is “1”, node *j* is not a constant; Dark red box = −3, means node *j* is co-expressed *oppositely* with node *j*; Red box = −2, means when node *i* is “0”, node *j* is not a constant, and node *i* is “1” results in *j* being “0”; Light red box = −1, means node *i* is “0” results in node *j* being “1”, and when node *i* is “1”, node *j* is not a constant; Yellow box = 5, means that the regulation is not clear. The white boxes indicate that the controlling genes have no effect on the corresponding column genes.

Since our goal for this study is to understand the regulation of CRPC, we did not use information on normal states in our network construction. The searching key words we used led only to references for CRPC. So, for the model constructed here, one cannot obtain the information on perturbations that may potentially able to switch the state from a cancer state to a normal state. Our intention for the study of the control effects of the genes is based on our understanding of the limitation of treatments. Usually, one can only target a few genes in a treatment, and it is thus beneficial for us to learn the control effects of the genes, since this could be used to select combinations of genes for targeting. We applied the approach described in the Materials and Methods section to compute the control effects of the gene in our CRPC network. The computed results are given in Fig. 3.

It can be seen from Fig. 3 that many genes in the MEK/ERK pathway, such as ERK, MED15, TGFB, MED1, UBE2C, FOXA1, JNK, CASP3, MAPK, IL-6, CCND, STAT3, JAK2, MMP-9, PI3K and IL-8, were positively controlled by many other genes, that is, when the corresponding controlling genes were “ON”, they would also be “ON”. These nodes play important roles in the progression of PCa. Some of them, such as UBE2C, CASP3, CCND1/2, STAT3, JAK2 and MMP-9, have been used as markers of cancer malignancy. Additionally, SKP2, Fkbp5, BCL-XL, WHSC1, and CDKN1B were also positively controlled by other genes. Note that the computed result shows that SOCS2, CDKN1A, E2F1, HOXB13, DAB2IP, IL-4, PDEF, BCL-2, PSA, NFKB, Androgen, mTOR, IKKa, CXCL1, CACNA1D, Bmi1, PAR1, NKX3.1, CDKN2A, FOXO1, SIRT1, ACK1, HSP27, HSP90, and EGF were hardly controlled by any single genes. This could be the result of only limited information on these genes is available currently, such as for the cases of NKX3.1, Bmi1, and CDKN2A.

We also found that there are 27 nodes, ADAM17, EGF, EGFR, RAF, MEK, ERK, Cdc 37, HSP90, MED15, SKIP, FHL2, NKX3.1, TGFB, Trap6, PAR1, Mark, Bmi1, AR, TNFa, CANA1D, IKKa, AKT, Wnt, DAB2IP, E2F1, CDKN1A, and IGF-1, played positive controlling roles in the network: if they were set to “ON”, the nodes controlled by them, such as EGFR, MED1, UBE2C, FOXA1, JNK, CASP3, MAPK, IL-6, PI3K, CCND, STAT3, JAK2, MMP-9, and IL-8 would become “ON”. On the other hand, CDKN2A, ERG, AT2R, PTEN, TMPRSS1, CACNA1D, and MTUS1 played negative controlling roles: if their expressions were set to “ON”, the nodes controlled by them would be “OFF”. Since ERK, MAPK, IL-6, and PI3K are important in negative feedback loops (compare with the discussions on feedback loops before), they could be potential targets for interrupting these negative feedback loops.

**Figure 4:**
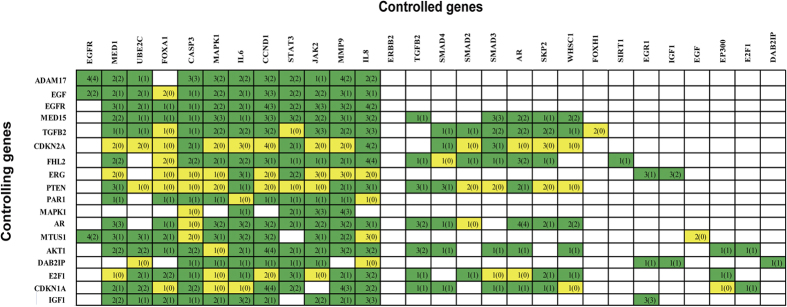
A comparison of the simulated control effects with the 4 datasets from GEO and the data from TCGA portal for some selected genes. “Controlling Genes” (18 genes) are labeled vertically; “Controlled Genes” (28 genes) are labeled horizontally. The effects on the Controlled Genes through the perturbation of each Controlling Gene are indicated by the corresponding row. Green color indicates that there is at least 1 dataset conformed to our controlling simulation and yellow color indicates that there are no dataset conformed with our controlling simulation. The number in each square grid indicates the consistent rate. For example, 4(3) means that there are 4 datasets showed obviously differences with Fold-change analysis, and among these 4 sets, 3 showed consistency with our controlling simulation.

To validate our simulation result on the control effects of genes, we searched expression datasets in GEO that were not included in the list of publications used for the construction of our network using the keywords: “androgen resistant” or “androgen independent” or “AR independent” or “AR resistant” or “castration-resistant” or “AR insensible” and “prostate cancer”. We selected 4 datasets (we required that each contains more than 10 samples) about CRPC for the validation. In addition, we also used prostate cancer sequence data from TCGA portal (http://cancergenome.nih.gov/) for our validation. The details of these 4 datasets are provided in Table 2. For each dataset, we separated the expressions of the aforementioned controlling genes into 2 levels: “high” and “low”, and compared the expression changes of all 91 target genes. If there existed several probes for a gene in a dataset, we used the one with the highest variation. We note that there would be a consistency rate of 77.99% if the requirement for consistency is that there are more than one datasets that conformed to our simulation result (Fig. 4).

### Experimental verification using FHL2 overexpression

**Figure 5:**
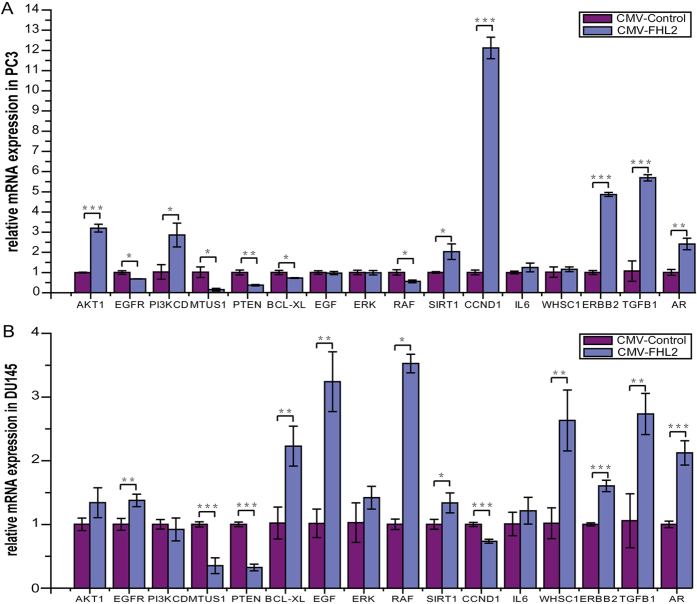
The expressions of 16 selected genes under FHL2 transfection on PC3 and DU145. The error bars depict the standard error of the mean of three replicates. The meanings of the asterisks: ***P < 0.001; **P < 0.01; *P < 0.05, two-tailed student’s t-test was used. (**A**) FHL2 overexpression and negative control in PC3 cell. (**B**) FHL2 overexpression and negative control in DU145.

Our computed results revealed that the expressions of ERK, RSK, ELK, TGFB, SMAD4, SMAD3, MED1, SIRT1, UBE2C, FOXA1, JNK, CASP3, MAPK, IL-6, CCND, TNFSF11, STAT3, JAK2, AR, c-Myc, MMP-9, PI3K, SKP2, Fkbp5, Bcl-XL, WHSC1, and IL-8 could be affected by FHL2 (Fig. 3). Since FHL2 was one of the genes that affect many genes according to our simulation and we had FHL2 over-expression plasmids in our lab at Guangxi Medical University, we chose FHL2 for experimental verification of our control simulation using overexpression. We chose ERK, TGFB1, TGFB2, SIRT1, IL-6, PI3K, and WHSC1 (which were controlled by several genes) and three other genes AR, BCL-XL, and CCND1 that could mark the deterioration of the cancer, for testing. Since PTEN, MTUS1, EGFR, RAF, EGF, ERBB2, and AKT play important roles on CRPC, we also verified their expression under FHL2 overexpression. The results were shown in Fig. 5.

The testing results showed that the overexpression of FHL2 induced the expressions of ERK, TGFB1, TGFB2, SIRT1, IL-6, WHSC1, and BCL-XL as predicted by our model. For DU145, the regulation of FHL2 on SIRT1 has been reported in the literature, but not for PC3. Our model predicted that FHL2 could increase the level of SIRT1 expression in PC3, which was confirmed by our experiment. Though PI3K and CCND1 showed lower expression levels in DU145, the absolute values of their fold changes were rather small.

The overexpression of FHL2 also affected the expressions of PTEN, MTUS1, EGFR, RAF, EGF, ERBB2, AKT, and AR. However, as will be discussed in the Materials and Methods section, a Boolean model’s partial control effects information can be recognized from the changes of node functions during simulations (partial controls would make the functions of the affected nodes simpler but did not reduce the functions to constants) and thus these effects were not shown in our computed results presented in Fig. 3.

## Discussion

We carried out a systematic study of CRPC regulation by constructing a comprehensive CRPC regulatory network, studying the topology of the constructed network, and analyzing an associated predictive Boolean model using novel methods. We hope to use the constructed network and its studies to provide guidance in our selection of gene targets, in particular multiple gene targets from different pathways, for the purpose of developing better treatments for CRPC in future studies.

The analysis that combining the detection with clustering analysis of all the stable states showed that the stable states of our Boolean model were naturally divided into two obvious groups, characterizing PC3 and DU145 cells respectively. This computed finding was consistent with the literature^22^,^24^,^25^,^26^,^27^,^28^. Our result suggested the possibility of using stable state analysis, such as clustering, as a new approach to derive information on the classification of complex diseases.

The control effect analysis of our Boolean model revealed new information for CRPC regulation system. It is known that MMP-9, JAK-STAT3, CCND1/2, and CASP3 are involved in many types of human cancers, and they are closely related with cell motility, invasion, and metastasis^29^,^30^,^31^,^32^. Figure 1 shows that they may be controlled by ERK1/2, MAPK/JNK, PI3K, IL-6, and IL-8 (partial results were reported in references^33^,^34^,^35^). Our Boolean model predicted the positive control effects of the imputing nodes ADAM17, MED15, SKIP, FHL2, Trap6 and IGF-1, and the negative control effects of MUST1, AT2R, PTEN and TMPRSS1 on these genes. The prediction implied that these upstream genes play important roles in the motility, invasion, and metastasis of prostate cancer cell via ERK1/2, MAPK/JNK, PI3K, IL-6, IL-8. The analysis of data from both GEO and TCGA portal supported our Boolean model simulation.

The gene AKT plays a key role in CRPC regulatory system. Our computed result based on our Boolean model suggested that no single gene could have a major impact on AKT’s expression, so it is desirable to discover more information on genes that can affect AKT. The results in references^33^,^34^,^35^,^36^ imply that AT2R is likely a participant in the neovascularization of CRPC. Now from our constructed network, one can see that MUTS1 is an activator of AT2R. So together, AT2R and MUTS1 could affect neovascularization and therefore affect AKT. However, our model suggested that MUTS1 and AT2R could affect AKT only in PC3 since the evidence to support this could only be found in cluster 2 and 3. Further study will be needed for confirmation.

It is known that LIM domains play an important role in protein-binding and FHL2 composes of four and a half LIM domains which make it possible for FHL2 to bind to multiple transcription protein in cancers^36^,^37^. Additionally, FHL2 is capable of shuttling between nucleus and cytoplasm. Some studies have identified significant nuclear accumulation of FHL2 in prostate cancer^38^,^39^, colon cancer^40^, and lung cancer^41^. These studies reported that FHL2 expression was absent in benign tissues, and that it would increase with disease progression and poor prognosis. In both our simulations and experiments, we found that the overexpression of FHL2 significantly increase the expression of RAF/ERK and PI3K/AKT pathways. It was reported in^42^ that FHL2 inhibits FOXO1 activity in prostate cancer cells by promoting the deacetylation of FOXO1 through SIRT1 on DU145. Our results imply the same should be true for PC3. In our experiment, we also found that the expressions of PTEN and MTUS1 were negatively affected by FHL2 overexpression. It was reported in^43^,^44^ that FOXO1 acts downstream on PTEN to induce the apoptosis of prostate cancer cells. Therefore, FHL2 may negatively affect the expression of PTEN. Finally, it was reported^45^,^46^ that FHL2 is correlated with neovascularization. These reports also showed that MTUS1 and AT2R might participate in neovascularization. Our study showed that MTUS1 was affected by FLH2 overexpression. All these strongly suggested that FLH2 plays a role in the regulation of the neovascularization of the cancer through regulating MTUS1.

Negative feedback loops are critical in biological systems^47^,^48^,^49^,^50^. For the constructed CRPC network, 119 positive feedback loops and 62 negative feedback loops were detected. It was found that AKT participated in each of these 62 negative feedback loops. Though a number of literature reported that the PI3K/AKT pathway is a key pathway for CRPC and could be a good target^51^,^52^ for new drugs, the role that AKT plays in the pathway is not clear from the existing literature. Our results suggested a further study of the role that AKT plays in CRPC regulation by investigating its roles in the feedback loops.

Future work includes carrying out further experimental studies of the predictions by our network and Boolean model, such as the control abilities of the nodes and the roles played by AKT and FHL2. Another possible direction for future work is to improve our network and model. The nodes in our study here include only genes and proteins. The integration of other molecules such as miRNA, hormone, and lnRNA could potentially improve the network and the model. Time scales are important factors in the modeling of a gene regulation system since the expressions of genes can change significantly depending on times. At the present, experimental data does not support a model of this size that also incorporates time as a variable. However, it is possible that we can use our study here as a guide to select smaller sets of the genes involved and then carry out a study of them using more detailed modeling schemes.

## Materials and Methods

### Constructing the CRPC signaling network

Our CRPC regulatory network was constructed based on literature mining. To obtain the needed information on the nodes to be included in the network and their interactions, we first searched the abstracts in PubMed by using the keywords: “androgen resistant”, “androgen independent”, “AR independent”, “AR resistant”, “castration-resistant”, “PC3”, “DU145”, and “prostate cancer”, and then verified the information further by consulting the original articles. We then assembled the information in a directed graph, in which the nodes are the genes or proteins (we will just call them “genes” for simplicity) and the edges describe the genes’ interactions^11^,^53^. Each interaction pair is connected with a directed edge from its source node (the upstream regulator) to the target node (the downstream regulator). Different end shapes of the directed edges were used to indicate whether the interaction is “promotes” or “inhibits”.

In order to make sure that the literature mining result is credible, we took three steps: First, two of the authors, HYL and YMZ, read the abstracts. If there existed a contradiction, HYL and YMZ discussed and read the corresponding papers carefully to resolve the issue. Second, after extracting the interaction information, we checked the included literature three more times to make sure that the information was correctly recorded. Third, We used our logical model to validate the network by comparing the computation result with the expression information available in Table S3. Except for Bmi1, the computed stable states showed consistency with the expression level information in Table S3, i.e. the expression level of a gene in the computed stable states showed the expected value accordingly.

### Constructing a Boolean model based on the CRPC network

We used the existing methods to translate the constructed CRPC network into a Boolean model^10^,^18^,^53^. However, due to the complexity of the network, there are various Boolean models that could fit the network. It should be noted that there are different models that are equally qualified unless a complete set of information is available. However, only partial information is known. Since we were interested in the long-term dynamical properties of CRPC regulation system, we selected our Boolean model based on statistical analyses of the computed stable states of the candidate models: the stable states of a good choice (a Boolean model) must represent the known expression information (as in the published data) of the genes well. The model we selected has a high consistency with known information on CRPC regulation. The selected model’s performance and our experiments further validated our selection.

### Computing and analyzing the stable states

We used a synchronous updating scheme in our computation. It is known that if flipping is not considered, then the set of stable states is the same whether the scheme is synchronous or asynchronous^4^. We applied the algorithm of^54^ to compute the stable states of a Boolean model. This algorithm was designed to handle Boolean models of complex biological systems such as gene regulatory networks. The idea behind the algorithm is that, for a Boolean model, the system of equations whose solutions are the stable states can be converted into a single equation; and a complex Boolean equation system, in particular that of a gene regulatory network, can be subdivided into smaller systems for the purpose of computing the solutions. In our CRPC Boolean model, there are 91 nodes. So the system of polynomial equations consists of 91 equations and 91 variables (see Table S5). Applying the algorithm, the system was divided into 6 subsystems. Since each subsystem was equivalent to a single equation, we first solved these 6 equations to obtain partial solutions and then patched these partial solutions to obtain all stable states according to the algorithm^54^. The statistical analyses were done using SAS.

### Computing the control effects of genes

To study the control effects of genes in a Boolean model, we developed a new computational method based on the polynomial representations of Boolean models. In the polynomial representation of a Boolean model, each node’s updating rule is given by a multivariate polynomial. In our Boolean model here, these polynomials can be denoted by 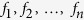, where *n* = *91*. For each node, we computed 4 sets that gave the control effects of the node. More precisely, for each node *k*, 1≤*k*≤*n* let *A(k,0)* (resp. *A(k,1)*) be the set of nodes that will eventually become 0 (resp. 1) via synchronous iterations after only node *k* is set to 0, and let *B(k,0)* (resp. *B(k,1)*) be the set of nodes that will eventually become 0 (resp. 1) after node *k* is set to 1. This can be done efficiently for all nodes at once. We computed these 4 sets by first substituting in 0 or 1 for the variable *x*_*k*_ (the variable that corresponds to node *k*), and then using iteration by replacing the nodes that had become constants (either 0 or 1) at each step. The iteration would stop when none of these four sets increased in size. The possible control effects of node *k* are provided by these 4 sets. For instance, the set *A(k,0)* gives the information on the nodes that are suppressed when node k is suppressed, and the set A(k,0) ∩ B(k,1) gives the information on the nodes that co-expressed with node *k*. Similarly, the set A(k,1) ∩ B(k,0) gives the information on the nodes that co-expressed *oppositely* with node *k*, etc. For our CRPC Boolean model, the total computation was done in a few seconds.

Since each node takes only values 0 and 1 in a Boolean model, the control effects computed for a Boolean model using the method described above do not specify the results on partial effects. The partial effects that are observed in experiments are reflected in a Boolean model through the changes of the node functions when certain nodes are set to 0 or 1. These changes do not reduce the functions to constants; they just change the update functions to different ones. To describe the partial effects between 0 and 1, different modeling methods that allow for more than two states for each node are needed. However, existing information does not support such a detailed model for the comprehensive CRPC network constructed here.

### Cell lines and cultures

Human PCa cell lines DU145 and PC3 were purchased from Shanghai Institutes of Biological Sciences of the Chinese Academy of Sciences (Shanghai, China). They were all cultured *in vitro* in DMEM/F12 supplemented with 10% fetal bovine serum (FBS; GIBCO) and incubated at 37°C in 5% CO2 at a sterile incubator.

### Construction of FHL2 overexpression plasmids and transfects

Full-length FHL2 cDNA was cloned into CMV-FHL2-EGFP-SV40-NEOMYCIN vector by GeneChem company (Shanghai, China) using primers (5′-CGCAAATGGGCGGTAGGCGTG-3′) and (5′-CGTCGCCGTCCAGCTCGACCAG-3′), and confirmed by a sequence analysis. According to the manufacturer’s protocol, DU145 and PC3 cells in good growth state were seeded into 6-well plates at a concentration of 5.5 × 10^6^ cells/well. When they reached 80–90% confluence, cells in the wells were transiently transfected with lipofectmine LTX transfection reagent (Invitrogen, USA). The transfection efficiency was assessed by the expression of GFP after incubating for 24 hours and 48 hours, respectively. qRT-PCR was applied to analyze the FHL2 mRNA levels.

### RNA isolation and quantification strategies in real-time PCR

After 48 hours, transfected cells were harvested. Total RNA was extracted using the Axygen RNA Isolation Kit (Axygen, USA). RNA concentration and quality were determined by absorbance ratio at 260:280 nm using a UV spectrophotometer. Then 1ug cDNA was reversely transcribed using a reverse transcription system (Invitrogen). Real-time PCR was performed with a 7500 Real-Time PCR system (Applied Biosystems, CA, USA) using SYBR Premix Ex TaqTM (Takara Bio).

Glyceraldehyde-3-phosphate dehydrogenase (GAPDH) expression level was used as normalization control. Relative expression values were calculated following the 2 −ΔΔ Ct method^54^. Primer sequences are listed in Table 3.

## Additional Information

**How to cite this article**: Hu, Y. *et al.* Integrated network model provides new insights into castration-resistant prostate cancer. *Sci. Rep.* **5**, 17280; doi: 10.1038/srep17280 (2015).

## Supplementary Material

Supplementary Information

## Figures and Tables

**Table 1:** The rates (frequencies) of “1” for 22 selected genes in each cluster of the stable states from Fig. 2A.

Genes	All	Clus1	Culs2	Clus3	Clus4	Group1 (clus1-3)	Group2 (Clus2-4)	Group1 – Group2	GSE41445 (LogFC)
Raf	0.219	0.290	0.930	0.013	0.672	0.875	0.197	0.678	2.000
CDKN2A	0.031	0.000	1.000	0.000	1.000	1.000	0.000	1.000	1.170
PTEN	0.500	0.465	1.000	0.524	1.000	1.000	0.484	0.516	7.810
NKX3.1	0.969	1.000	0.000	1.000	0.000	0.000	1.000	−1.000	2.730
Bmi1	0.969	1.000	0.000	1.000	0.000	0.000	1.000	−1.000	0.979
TNFa	0.969	1.000	0.000	1.000	0.000	0.000	1.000	−1.000	//
EBP1	0.500	0.538	1.000	0.370	1.000	1.000	0.484	0.516	0.742
CXCL1	0.250	0.325	1.000	0.016	1.000	1.000	0.226	0.774	1.170
BMP-6	0.250	0.324	1.000	0.016	1.000	1.000	0.226	0.774	0.334
IKKa	0.750	0.675	0.000	0.983	0.000	0.000	0.774	−0.774	//
AKT	0.750	0.675	0.000	0.984	0.000	0.000	0.774	−0.774	1.972
mTOR	0.750	0.675	0.000	0.984	0.000	0.000	0.774	−0.774	2.210
NFKB	0.250	0.325	1.000	0.016	1.000	1.000	0.226	0.774	1.590
INPP4B	0.500	0.585	1.000	0.269	1.000	1.000	0.484	0.516	0.306
BCL-2	0.250	0.324	1.000	0.016	1.000	1.000	0.226	0.774	0.355
NCOR	0.500	0.585	1.000	0.269	1.000	1.000	0.484	0.516	0.703
ZnT4	0.500	0.548	0.000	0.448	0.000	0.000	0.516	−0.516	//
HOXB13	0.500	0.451	1.000	0.551	1.000	1.000	0.484	0.516	−8.060
E2F1	0.937	0.953	0.000	0.997	0.000	0.000	0.968	−0.968	−1.130
CDKN1A	0.750	0.806	0.000	0.705	0.000	0.000	0.774	−0.774	//
CK2	0.500	0.415	0.000	0.731	0.000	0.000	0.516	−0.516	//
RAC1	0.500	0.464	1.000	0.534	1.000	1.000	0.484	0.516	0.142

These 22 genes are the genes that showed significant difference between Group 1 (clusters 1 and 4) and Group 2 (clusters 2 and 3) from Fig. 2A. A gene was selected if the absolute value of the difference between its expressed rates (showed “1”) in Group 1 and in Group 2 is >0.5. The last column denotes the logFC value of DU145 cells vs. PC3 cells on these 22 genes’ expressions.

**Table 2:** Information of the 4 expression datasets from GEO and the data from TCGA portal.

Series ID	Platform	Sample number	Experiment material	Phenotype
GSE2443	Affymetrix Human Genome U133A Array	10	Tissue (Homo sapiens)	Androgen-independent
GSE29650	Illumina HumanHT-12 V3.0 expression beadchip	30	Bone metastases (Homo sapiens)	Castration-resistant
GSE32269	Affymetrix Human Genome U133A Array	30	Bone metastases (Homo sapiens)	Castration-resistant
TCGA (PCa)	Illuminahiseq_rnaseqv2	10	Tissue (Homo sapiens)	No response to drugs

**Table 3:** Genes and their primer sequences from qRT-PCR.

Genes	Forward	Reverse
FHL2	5′-CCAAGTGCCAGGAATGCAAG-3′	5′-TCTCATAGCAGGGCACACAGAA-3′
PTEN	5′-GAGCGTGCAGATAATGACAAGGAAT-3′	5′-GGATTTGACGGCTCCTCTACTGTTT-3′
MTUS1	5′-CAAATTGAAGCGTTTCCAGCAG-3′	5′-CCATTGTGCAGTTTCCACAGAAG-3′
SIRT1	5′-CCCAGAACATAGACACGCTGGA-3′	5′-ATCAGCTGGGCACCTAGGACA-3′
PI3K	5′-TTCAACAAGGATGCCCTGCTC-3′	5′-GGATCATGATGTTGTCGCTGTG-3′
EGFR	5′-CATCCAGGCCCAACTGTGAG-3′	5′-CAGTGGAAGCCTTGAAGCAGAA-3′
WHSC1	5′-TTCTGCACCAAGGCCTACCAC-3′	5′-AGGTTTGCCACACACGTCACA -3′
BCL-XL	5′-CTGGCTCCCATGACCATACTGA-3′	5′-GTGAGGCAGCTGAGGCCATAA -3′
RAF	5′-ACACCCAGAGGAGCACATCAGA-3′	5′-ACACCCAGAGGAGCACATCAGA -3′
EGF	5′-GCACGTGCCCTGTAGGATTTG-3′	5′-AGACACATTGCGTGGACAGGA -3′
ERK	5′-CGTTGGTACAGGGCTCCAGAA-3′	5′-CTGCCAGAATGCAGCCTACAGA-3′
ERBB2	5′-TGGCACAGTCTACAAGGGCATC-3′	5′-TGGCACAGTCTACAAGGGCATC-3′
TGFB1	5′-CGCATCCTAGACCCTTTCTCCTC-3′	5′-GGTGTCTCAGTATCCCACGGAAAT-3′
TGFB2	5′-TGGATGCGGCCTATTGCTTTA-3′	5′-CCAGCACAGAAGTTGGCATTGTA -3′
EBP1	5′-GCCAGAGCTGTGCAGATGAG-3′	5′-TCAGCAGGCTGGCATTTG-3′
E2F1	5′-TGCTCTCCGAGGACACTGAC-3′	5′-ATCGGGCCTTGTTTGCTCTT-3′
Bmi1	5′-CTGCAGCTCGCTTCAAGATG-3′	5′-TTAGCTCAGTGATCTTGATTCTCGT-3′
CDKN2A	5′-TGAGGCGCCCTTTGGTTATC-3′	5′-GAGGTTTCTCAGAGCCTCTCTGGT-3′
GADPH	5′-AACGGATTTGGTCGTATTG-3′	5′-CTGGAAGATGGTGATGGG -3′
