# Supplementary material for: Integrated network model provides new insights into castration-resistant prostate cancer
Source: Sci Rep. 2015 Nov 25;5:17280. doi: 10.1038/srep17280 (PMC4658549; doi:10.1038/srep17280)
Supplement: Supplementary Information [file srep17280-s1.doc]

**Supplementary Information for**

Integrated network model provides new insights into castration-resistant prostate cancer

Yanling Hu^1,2^ , Yinmin Gu^1^ , Huimin Wang ^1^ , Yuanjie Huang^1^ , and Yi Ming Zou ^3*^

^1^Experimental Centre of Medical Sciences, Guangxi Medical University, Nanning, Guangxi, 530021, China

^2^Center for Genomic and Personalized Medicine, Guangxi Medical University, Nanning, Guangxi, 530021, China

^3^Department of Mathematical Sciences, University of Wisconsin-Milwaukee, Milwaukee, WI, 53201, USA

Yi Ming Zou: [ymzou@uwm.edu](mailto:ymzou@uwm.edu)

* To whom correspondence should be addressed. Tel: 414-229-5110; Fax: 414-229-4907; Email: ymzou@uwm.edu

**Fig S1. Nodes’ participations in the feedback loops.** The darker the color, the more feedback loops of a node participates in. Colorless nodes are not in any of the feedback loops. Together, the colored nodes form a strongly connected component in the graph.

**Table S1.** Genes and interaction information from the selected 119 references among the 5,115 abstracts found through PubMed.

**Table S2.** Full names and the abbreviations of the genes in Table S1.

**Table S3.** Known deregulations of the genes in CRPC network.

**Table S4.** The 62 detected negative feed-back loops.

**Table S5.** Polynomial functions of the nodes of the Boolean model.

**Fig S1.**
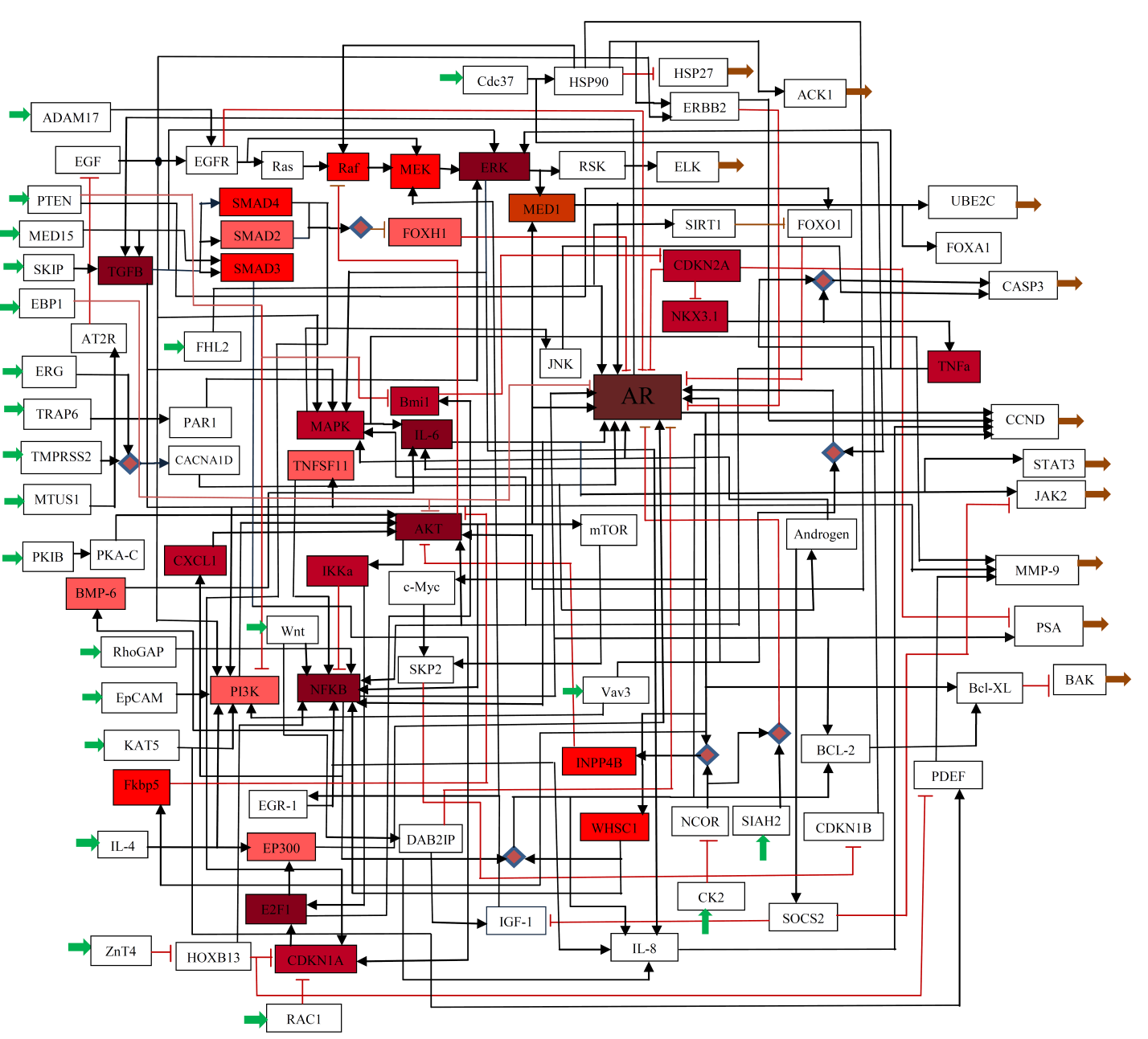


Table S1.

| source(upstream) node | target(downstream) node | Interation | direct interaction | Ref |
| --- | --- | --- | --- | --- |
| NFKB | PSA | activation | direction | 1 |
| AR | c-Myc | activation | direction | 1 |
| NFKB | BCL2 | Activation | indirection | 2 |
| TGFB | TNFSF11 | induced | direction | 2 |
| Rac1 | CDKN1A | suppress | indirection | 3 |
| RSK | Elk | activation | direction | 4 |
| Raf | MEK | activation | direction | 4 |
| ERK | RSK | activation | direction | 4 |
| EGF | PI3K | activation | direction | 5 |
| IL-4 | PI3K | activation | indirection | 6 |
| FHL2 | SIRT1 | promoting | direction | 7 |
| FKBP51 | AR | activation | direct | 8 |
| PAR1 | TRAP6 | activating | direction | 9 |
| PAR1 | ERK | activation |  | 9 |
| ERK | MAPK | activation | direction | 9 |
| TRAP6 | PAR1 | INDUCE | indirection | 9 |
| FoxH1 | AR | repressed | direction | 10 |
| Smad2, Smad4 | FoxH1 | relieved | direction | 10 |
| Hsp90 | Ack1 | activation | direction | 11 |
| Hsp27 | Stat3 | inhibition | not sure | 12 |
| RGS2 | AR | inhibition | not sure | 13 |
| AKT | AR | activation | direction | 14 |
| Bcl-xL | Bak | inhibition | direction | 15 |
| PTEN | Raf | Activation | direction | 16 |
| Akt | Raf | suppress | direction | 16 |
| FGF | Spry | activation | direct | 17 |
| SIAH2 | Spry1 | degradation | direct | 17 |
| SIAH2 | Spry2 | degradation | direct | 17 |
| SIAH2,Spry2 | FGF2 | rescue | direct | 17 |
| AR | CCND1 | activation | not sure | 18 |
| AR | CCNB | activation | not sure | 18 |
| AR | CDK1 | activation | not sure | 18 |
| NCOA3 | cdk2 | activation | not sure | 18 |
| NCOA3 | CCNE | activation | not sure | 18 |
| AR | CCND | Activation | indirection | 18 |
| NCOA3 | CCNE | Activation | indirection | 18 |
| SAPKs | IL-6 | activation | not sure | 19 |
| MAPK | IL-6 | activation |  | 19 |
| ErbB-2 | CCND | induced | direction | 20 |
| Nkx3.1 | Bax | promoted | indirection | 21 |
| Nkx3.1 | Bcl-2 | promoted | indirection | 21 |
| Nkx3.1 | CASP3 | promoted | indirection | 21 |
| ERK | IL-8 | stimulates | direction | 22 |
| IGF-1 | MEK | stimulated | direction | 22 |
| IL-8 | CCND | induced | indirection | 23 |
| androgen | AR | activation | direction | 24 |
| AR | Bcl-xL | stimulated | direction | 24 |
| HSP90 | AKT | Activation | indirection | 25 |
| HSP90 | CRAF | Activation | indirection | 25 |
| HSP90 | ErbB2 | Activation | indirection | 25 |
| EGF | PGE2 | activation | not sure | 26 |
| Bmi1 | CDKN2A | suppressed | indirection | 27 |
| EP300 | AR | activation | direction | 28 |
| IL-4 | EP300 | activation | direction | 28 |
| EBP1 | AKT | inhibition | indirection | 29 |
| AR | EBP1 | corepressor | indirection | 29 |
| EGFR | Ras | Activation | indirection | 30 |
| Ras | Raf | Activation | direction | 30 |
| Nkx3.1, CDKN1B | CASP3 | activation | indirection | 31 |
| AR | WHSC1 | recruited | indirection | 32 |
| PKA-C | AKT | Activation | direction | 33 |
| PKIB | PKA-C | Activation | direction | 33 |
| ErbB2 | AR | inhibition | indirection | 34 |
| EGFR | AR | inhibition | indirection | 34 |
| EGF | ErbB2 | activation | direction | 34 |
| AMACR | BTG1 | inhibition | not sure | 35 |
| AMACR | IGF-1 | inhibition | not sure | 35 |
| AMACR | AR | inhibition | not sure | 35 |
| SOCS-3 | Bcl-2 | activation | not sure | 36 |
| EGR-1 | IL-8 | activation | direction | 37 |
| EGR-1 | NFKB | activation | direction | 37 |
| NFKB | IL-8 | activation | indirection | 37 |
| PTEN | Bmi1 | inhibition | direction | 38 |
| MAPK | ERK | activation | direction | 39 |
| IKKB | IKKa | Activation | indirection | 40 |
| IKKB | Stat3 | Activation | indirection | 40 |
| HOXB13 | CDKN1A | inhibition | indirection | 41 |
| CDKN1A | E2F1 | promotion | indirection | 41 |
| AR | ErbB3 | Inhibition | not sure | 42 |
| IKKα | cytokines | Activation | direct | 42 |
| cytokines | STAT3 | Activation | direct | 42 |
| cytokines | IKKα | Activation | direct | 42 |
| NKX3.1 | cycloheximide | inducer | not sure | 43 |
| NKX3.1 | TNFa | promote | indirection | 43 |
| MTUS1 | AT2R | activation | indirection | 44 |
| AT2R | EGF | inhibition | indirection | 44 |
| ECE-1 | PTK2 | activation | direct | 45 |
| PMA | ST3Gal | Activation | not sure | 46 |
| INPP4B | AKT | Inhibition | direction | 47 |
| AR, NCoR | INPP4B | induction | indirection | 47 |
| SMAD3 | CDKN1A | promote | indirection | 48 |
| SMAD4 | CDKN1A | promote | indirection | 48 |
| TGFB | PMEPA1 | induced | indirection | 48 |
| PMEPA1 | SMAD3 | suppress | direction | 48 |
| PMEPA1 | CDKN1A | suppress | indirection | 48 |
| TPD52 | Bcl-2 | activation | not sure | 49 |
| MED1 | FOXA1 | Activation | indirection | 50 |
| MED1 | UBE2C | recruitment | direction | 50 |
| sGCα1 | TP53 | Inhibition | direct | 51 |
| EGFR | MEK | activation | direct | 52 |
| MEK | ERK | activation | direct | 52 |
| ADAM17 | EGFR | activation | indirection | 52 |
| EGFR | MEK | activation | direction | 52 |
| TNFSF11 | Mcl-1 | Activation | direct | 53 |
| TNFSF11 | NFKB | Activation | direction | 53 |
| IL-6 | JAK2 | Activation | direction | 54 |
| IL-6 | Stat3 | Activation | direction | 54 |
| TGFBR1 | Smad2 | activation | direct | 55 |
| TGFBR1 | Smad3 | activation | direct | 55 |
| TGFB | Smad2 | induced | direction | 55 |
| TGFB | Smad3 | induced | direction | 55 |
| c-Myc | Skp2 | activation | direction | 56 |
| androgen | PTPN1 | activation | not sure | 57 |
| AR | TACC2 | inhibition | indirection | 58 |
| WHSC1，NFKB | VEGFA | activation | not sure | 59 |
| WHSC1，NFKB | Bcl-2 | activation | indirection | 59 |
| WHSC1，NFKB | CCND | activation | indirection | 59 |
| WHSC1，NFKB | IL-6 | activation | indirection | 59 |
| WHSC1，NFKB | IL-8 | activation | indirection | 59 |
| WHSC1 | NFKB | coactivator | direction | 59 |
| IL-6 | NFKB | induced | direction | 59 |
| NFKB | WHSC1 | coactivator | direction | 59 |
| MAPK | paxillin | Activation | not sure | 60 |
| MAPK | paxillin | Activation | not sure | 60 |
| androgen | MAPK | promoted | direction | 60 |
| EGF | MAPK | promoted | direction | 60 |
| PTEN | PI3K | inhibition | indirection | 61 |
| PAGE4 | AR | inhibition | direct | 62 |
| JNK | CASP3 | activation | direction | 63 |
| MAPK | JNK | activation | direction | 63 |
| TGFB | MAPK | activation | direction | 63 |
| KLF8 | AR | co-activator | direct | 64 |
| activin A | AKR1C3 | inhibition | not sure | 65 |
| CXCL1 | AKT | Activation | indirection | 66 |
| AKT | IKKA | activation | direction | 66 |
| NFKB | CXCL1 | Activation | direction | 67 |
| Cdc37 | Hsp90 | co-chaperone | direct | 68 |
| Cdc37,vav3 | AR | activation | direction | 68 |
| Cdc37 | Hsp90 | co-chaperone | direction | 68 |
| AR | NOV | inhibition | direct | 69 |
| AR | EZH2 | recruits | direct | 69 |
| EZH2 | NOV | inhibition | direct | 69 |
| AR | Sox2 | inhibition | direct | 70 |
| KLLN | PSA | inhibition | not sure | 71 |
| KLLN | AR | inhibition | not sure | 71 |
| KLLN | TP53 | activation | not sure | 71 |
| KLLN | TP73 | activation | not sure | 71 |
| FOXO1 | AR | inhibition | direction | 72 |
| PTEN | FOXO1 | Activation | indirection | 72 |
| CDKN2A | AR | corespressor | direction | 73 |
| !AR | CDKN2A | corespressor | direction | 73 |
| CDKN2A | NKX3.1 | suppress | indirection | 73 |
| CDKN2A | PSA | Suppress | indirection | 73 |
| TGF-β | PI3K | activation | indirection | 74 |
| AR | TGFB | activation | indirection | 75 |
| MED1 | AR | coactivator | direction | 76 |
| AR | MED1 | coactivator | direction | 76 |
| Nkx3.1, Pten | MED1 | markedly reduced | indirection | 76 |
| ERK | MED1 | promoted | indirection | 76 |
| AKT | MED1 | promoted | indirection | 76 |
| DAB2IP | AR | inhibition | indirection | 77 |
| Fkbp5 | AKT | inhibition | indirection | 78 |
| androgen | CCNE | repress | indirection | 78 |
| AR | Fkbp5 | Response | direction | 78 |
| CK2,NCoR | IP-10 | inhibition | not sure | 79 |
| CK2 | NCoR | inhibition | direction | 79 |
| BMP-6 | IL-6 | activation | indirection | 80 |
| TNFa | AKT | activation | indirection | 81 |
| TNFa | ERK | activation | indirection | 81 |
| TNFa | MAPK | activation | indirection | 81 |
| TNFa | NFKB | activation | indirection | 81 |
| E2F1 | Bmi1 | recruitment | direction | 82 |
| E2F1 | EP300 | coactivator | direction | 82 |
| IKKa | E2F1 | promotes | direction | 82 |
| FHL2 | AR | coactivation | direction | 83 |
| AR | FHL2 | coactivation | direction | 83 |
| Wnt | DAB2IP | Activation | indirection | 84 |
| IGF-1 | Egr-1 | activation | indirection | 84 |
| DAB2IP | IGF-1 | Activation | indirection | 84 |
| IKKa | NFKB | inhibition | indirection | 85 |
| IL-6 | NR1I2 | activation | direct | 86 |
| UBIAD1 | cholesterol | inhibition | not sure | 86 |
| GDIα | AR | inhibition | direct | 87 |
| SREBP-1 | AR | induced | not sure | 88 |
| SOCS2 | Src | inhibition | not sure | 89 |
| SOCS2 | STAT5 | inhibition | direction | 89 |
| SOCS2 | IGF-1 | suppress | indirection | 89 |
| SOCS2 | JAK2 | inhibition | indirection | 89 |
| Androgen | SOCS2 | induce | indirection | 89 |
| LAT | TCF-4 | Inhibition | not sure | 90 |
| LAT | ASCT1 | Inhibition | direct | 90 |
| LAT | ASCT2 | Inhibition | direct | 90 |
| CACNA1D | androgen | Activation | indirection | 91 |
| CACNA1D | AR | Activation | indirection | 91 |
| TMPRSS2, ERG | CACNA1D | significantly higher | direction | 91 |
| Bcl-2 | Bcl-xL | activation | direction | 92 |
| EpCAM | 4EBP1 | activation | not sure | 93 |
| EpCAM | S6K | activation | not sure | 93 |
| EpCAM | E-cadherin | activation | not sure | 93 |
| EpCAM | PI3K | activation | indirection | 93 |
| ZnT4 | HOXB13 | Inhibition | indirection | 94 |
| HOXB13 | NFKB | stimulating | indirection | 94 |
| TGFB | ERK | activation | indirection | 95 |
| MAPK | mmp-9 | Activation | indirection | 95 |
| SKIP | TGFB | activation | indirection | 95 |
| FOXA1 | Wnt | enriched | indirection | 96 |
| FOXA1 | PTEN | enriched | indirection | 96 |
| KAT5 | PDEF | activation | indirection | 97 |
| KAT5 | PI3K | activation | indirection | 97 |
| PI3K | mTOR | Activation | direction | 98 |
| mTOR | Skp2 | activation | indirection | 98 |
| MED15 | Smad3 | activation | direction | 99 |
| MED15 | TGFB | activation | direction | 99 |
| Hsp90 | TF6 | activation | not sure | 100 |
| Hsp90 | GRP78 | activation | not sure | 100 |
| Hsp90 | DDIT3 | activation | not sure | 100 |
| !Hsp90 | Hsp27 | inhibition | indirection | 100 |
| Slzip,CCND3 | AR | represses | direct interaction | 101 |
| Nrf2 | AR | inhibition | not sure | 102 |
| p120,Nrf1 | AR | suppress | not sure | 102 |
| ep300, pten | DAB2IP | indution | indirection | 103 |
| FGF9 | VEGFA | indution | not sure | 104 |
| FGF9 | N-cadherin | indution | not sure | 104 |
| NFKB | BMP-6 | activation | indirection | 105 |
| WNT5A | NFKB | Activation | indirection | 105 |
| RhoGAP | Cdc42 | inhibition | not sure | 106 |
| RhoGAP | IKBA | inhibition | direction | 106 |
| RhoGAP | NFKB | Activation | direction | 106 |
| PDEF | MMP-9 | activation | indirection | 107 |
| HOXB13 | PDEF | inhibition | indirection | 107 |
| MEK | ERK | activation | direction | 4,22 |
| PI3K | AKT | activation | direction | 6, 108, 109, 61, 93 |
| NFKB | AR | activation | indirection | 6, 110, 111 |
| AKT | NFKB | activation | indirection | 6, 66 |
| !SIRT1 | FOXO1 | inhibit | direction | 7，112 |
| Vav3 | PI3K | activation | direction | 14, 109 |
| EBP1 | AR | corepressor | indirection | 29, 113 |
| EGF | EGFR | activation | direction | 34, 5 |
| Vav3 | AR | coactivates | direction | 114,115 |
| Akt | mTOR | Activation | direction | 61, 93 |
| Siah2,NCOR1 | AR | inactivation | indirection | 116 |
| IL-6 | AR | activation | direction | 75, 80 |
| TGFB | MMP-9 | activation | indirection | 75, 117, 95 |
| AKT | MED1 | promoted | indirection | 76, 118 |
| !Skp2 | CDKN1B | block | direction | 118, 119 |
| FOXA1 | PTEN | enriched | indirection | 96, 98 |

**REFERENCE**

1. Chen, C.D. & Sawyers, C.L. NF-kappa B activates prostate-specific antigen expression and is upregulated in androgen-independent prostate cancer. *Mol Cell Biol.* **22**, 2862-2870 (2002).

2. Suh, J. & Rabson, A.B. NF-kappaB activation in human prostate cancer: important mediator or epiphenomenon? *J Cell Biochem.* **91**, 100-117 (2004).

3. Knight-Krajewski, S. *et al*. Deregulation of the Rho GTPase, Rac1, suppresses cyclin-dependent kinase inhibitor p21(CIP1) levels in androgen-independent human prostate cancer cells. *Oncogene.* **23**, 5513-5522 (2004).

4. Koochekpour, S. *et al*. Prosaptide TX14A stimulates growth, migration, and invasion and activates the Raf-MEK-ERK-RSK-Elk-1 signaling pathway in prostate cancer cells, *Prostate.* **61**: 114-123 (2004).

5. Bonaccorsi, L. *et al*. EGF receptor (EGFR) signaling promoting invasion is disrupted in androgen-sensitive prostate cancer cells by an interaction between EGFR and androgen receptor (AR). *Int J Cancer.* **112**, 78-86 (2004).

6. Lee, S.O., Lou, W., Nadiminty, N., Lin, X. & Gao, A.C. Requirement for NF-(kappa)B in interleukin-4-induced androgen receptor activation in prostate cancer cells. *Prostate.* **64**, 160-167 (2005).

7. Yang, Y., Hou, H., Haller, E.M., Nicosia, S.V. & Bai, W. Suppression of FOXO1 activity by FHL2 through SIRT1-mediated deacetylation. *EMBO J.* **24**, 1021-1032 (2005).

8. Febbo, P.G. *et al*. Androgen mediated regulation and functional implications of fkbp51 expression in prostate cancer. *J Urol.* ***173***, 1772-17727 (2005).

9. Tantivejkul, K. *et al*. PAR1-mediated NFkappaB activation promotes survival of prostate cancer cells through a Bcl-xL-dependent mechanism. *J Cell Biochem.* **96**, 641-652 (2005).

10. Chen, G. *et al*. Modulation of androgen receptor transactivation by FoxH1. A newly identified androgen receptor corepressor. *J Biol Chem.* **280,** 36355-36363 (2005).

11. Mahajan, N.P., Whang, Y.E., Mohler, J.L. & Earp, H.S. Activated tyrosine kinase Ack1 promotes prostate tumorigenesis: role of Ack1 in polyubiquitination of tumor suppressor Wwox. *Cancer Res.* **65**, 10514-10523 (2005).

12. Rocchi P. *et al*. Increased Hsp27 after androgen ablation facilitates androgen-independent progression in prostate cancer via signal transducers and activators of transcription 3-mediated suppression of apoptosis. *Cancer Res.* **65**, 11083-11093 (2005).

13. Cao, X. *et al*. Regulator of G-protein signaling 2 (RGS2) inhibits androgen-independent activation of androgen receptor in prostate cancer cells. *Oncogene.* **25**, 3719-3734 (2006).

14. Dong, Z. *et al*. Vav3 oncogene is overexpressed and regulates cell growth and androgen receptor activity in human prostate cancer. *Mol Endocrinol.* **20**, 2315-2325 (2006).

15. Castilla, C. *et al*. Bcl-xL is overexpressed in hormone-resistant prostate cancer and promotes survival of LNCaP cells via interaction with proapoptotic Bak. *Endocrinology.* **147**, 4960-4967 (2006).

16. McCubrey, J.A. *et al*. Roles of the RAF/MEK/ERK and PI3K/PTEN/AKT pathways in malignant transformation and drug resistance. *Adv Enzyme Regul.* **46**, 249-279 (2006).

17. Nadeau, R.J., Toher, J.L., Yang, X., Kovalenko, D. & Friesel, R. Regulation of Sprouty2 stability by mammalian Seven-in-Absentia homolog 2. *J Cell Biochem.* **100**, 151-160 (2007).

18. Zou, J.X. *et al*. ACTR/AIB1/SRC-3 and androgen receptor control prostate cancer cell proliferation and tumor growth through direct control of cell cycle genes. *Prostate.* **66**, 1474-1486 (2006).

19. Shida, Y., Igawa, T., Hakariya, T., Sakai, H. & Kanetake, H. p38MAPK activation is involved in androgen-independent proliferation of human prostate cancer cells by regulating IL-6 secretion. *Biochem Biophys Res Commun.* **353**, 744-749 (2007).

20. Casimiro, M. *et al*. ErbB-2 induces the cyclin D1 gene in prostate epithelial cells in vitro and in vivo. *Cancer Res****.* 67**, 4364-4372 (2007).

21. Wang, P. *et al*. Expression of Nkx3.1 enhances 17beta-estradiol anti-tumor action in PC3 human prostate cancer cells. *Asian J Androl.* **9**, 353-360 (2007).

22. Kooijman, R., Himpe, E., Potikanond, S. & Coppens, A. Regulation of interleukin-8 expression in human prostate cancer cells by insulin-like growth factor-I and inflammatory cytokines. *Growth Horm IGF Res**.* **17**, 383-391 (2007).

23. MacManus. C.F. *et al*. Interleukin-8 signaling promotes translational regulation of cyclin D in androgen-independent prostate cancer cells. *Mol Cancer Res****.* 5**, 737-748 (2007).

24. Sun, A. *et al*. Androgen receptor-dependent regulation of Bcl-xL expression: Implication in prostate cancer progression. *Prostate.* **68**, 453-461 (2008).

25. Eccles, S.A. *et al*. NVP-AUY922: a novel heat shock protein 90 inhibitor active against xenograft tumor growth, angiogenesis, and metastasis. *Cancer Res.* **68**, 2850-2860 (2008).

26. Jia, R.P. *et al*. Cyclooxygenase-2 expression is dependent upon epidermal growth factor receptor expression or activation in androgen independent prostate cancer. *Asian J Androl.* **10**, 758-764 (2008).

27. Fan, C. *et al*. Bmi1 promotes prostate tumorigenesis via inhibiting p16(INK4A) and p14(ARF) expression. *Biochim Biophys Acta.* **1782**, 642-8 (2008).

28. Lee, S.O. *et al*. Interleukin-4 activates androgen receptor through CBP/p300. ***Prostate* 69**, 126-132 (2009).

29. Zhang, Y. *et al*. EBP1, an ErbB3-binding protein, is decreased in prostate cancer and implicated in hormone resistance. *Mol Cancer Ther.* **7**, 3176-3186 (2008).

30. Bouali, S. *et al*. PTEN expression controls cellular response to cetuximab by mediating PI3K/AKT and RAS/RAF/MAPK downstream signaling in KRAS wild-type, hormone refractory prostate cancer cells. *Oncol Rep.* **21**, 731-735 (2009).

31. Wang, P. *et al*. Nkx3.1 and p27(KIP1) cooperate in proliferation inhibition and apoptosis induction in human androgen-independent prostate cancer cells. *Cancer Invest.* **27**, 369-375 (2009).

32. Kang, H.B. *et al*. The histone methyltransferase, NSD2, enhances androgen receptor-mediated transcription. *FEBS Lett.* **583**, 1880-1886 (2009).

33. Chung, S. *et al*. Overexpressing PKIB in prostate cancer promotes its aggressiveness by linking between PKA and Akt pathways. *Oncogene.* **28**, 2849-2859 (2009).

34. Cai, C. *et al*. Androgen receptor expression in prostate cancer cells is suppressed by activation of epidermal growth factor receptor and ErbB2. *Cancer Res.* **69**, 5202-5209 (2009).

35. Takahara, K. et al. Conversion of prostate cancer from hormone independency to dependency due to AMACR inhibition: involvement of increased AR expression and decreased IGF1 expression. *Anticancer Res.* **29**, 2497-2505 (2009).

36. Puhr, M. *et al*. Down-regulation of suppressor of cytokine signaling-3 causes prostate cancer cell death through activation of the extrinsic and intrinsic apoptosis pathways. *Cancer Res.* **69**, 7375-7384 (2009).

37. Ma, J. *et al*. Targeted knockdown of EGR-1 inhibits IL-8 production and IL-8-mediated invasion of prostate cancer cells through suppressing EGR-1/NF-kappaB synergy. *J Biol Chem.* **284**, 34600-34606 (2009).

38. Fan, C. *et al*. PTEN inhibits BMI1 function independently of its phosphatase activity. *Mol Cancer.* **8**, 98 (2009).

39. Wegiel, B. *et al*. Cystatin C is downregulated in prostate cancer and modulates invasion of prostate cancer cells via MAPK/Erk and androgen receptor pathways. *PLoS One.* **4**, e7953 (2009).

40. Ammirante, M., Luo, J.L., Grivennikov, S., Nedospasov, S. & Karin, M. B-cell-derived lymphotoxin promotes castration-resistant prostate cancer. *Nature.* **464**, 302-305 (2010).

41. Kim, Y.R. *et al*. HOXB13 promotes androgen independent growth of LNCaP prostate cancer cells by the activation of E2F signaling. *Mol Cancer.* **9**, 124 (2010).

42. Chen, L. *et al*. Nrdp1-mediated regulation of ErbB3 expression by the androgen receptor in androgen-dependent but not castrate-resistant prostate cancer cells. *Cancer Res.* **70**, 5994-6003 (2010).

43. Pengju, Z. *et al*. NKX3.1 potentiates TNF-alpha/CHX-induced apoptosis of prostate cancer cells through increasing caspase-3 expression and its activity. *Biochem Biophys Res Commun.* **398**, 457-461 (2010).

44. Louis, S.N. *et al*. Expression and function of ATIP/MTUS1 in human prostate cancer cell lines. *Prostate.* **70**, 1563-1574 (2010).

45. Whyteside, A.R., Hinsley, E.E., Lambert, L.A., McDermott, P.J. & Turner, A.J. ECE-1 influences prostate cancer cell invasion via ET-1-mediated FAK phosphorylation and ET-1-independent mechanisms. *Can J Physiol Pharmacol .* **88**, 850-854 (2010).

46. Hatano, K., Miyamoto, Y., Nonomura, N. & Kaneda, Y. Expression of gangliosides, GD1a, and sialyl paragloboside is regulated by NF-κB-dependent transcriptional control of α2,3-sialyltransferase I, II, and VI in human castration-resistant prostate cancer cells. *Int J Cancer.* **129**, 1838-1847 (2011).

47. Hodgson, M.C. *et al*. Decreased expression and androgen regulation of the tumor suppressor gene INPP4B in prostate cancer. *Cancer Res* ***.* 71**, 572-582 (2011).

48. Liu, R., Zhou, Z., Huang, J. & Chen, C. PMEPA1 promotes androgen receptor-negative prostate cell proliferation through suppressing the Smad3/4-c-Myc-p21 Cip1 signaling pathway. *J Pathol.* **223**, 683-694.

49. Zhang, D. *et al*. PrLZ protects prostate cancer cells from apoptosis induced by androgen deprivation via the activation of Stat3/Bcl-2 pathway. *Cancer Res.* **71**, 2193-2202 (2011).

50. Chen, Z. *et al*. Phospho-MED1-enhanced UBE2C locus looping drives castration-resistant prostate cancer growth. *EMBO J.* **30**, 2405-2419 (2011).

51. Cai, C. *et al*. Soluble guanylyl cyclase α1 and p53 cytoplasmic sequestration and down-regulation in prostate cancer. *Mol Endocrinol.* **26**, 292-307 (2012).

52. Xiao, L.J. *et al*. ADAM17 targets MMP-2 and MMP-9 via EGFR-MEK-ERK pathway activation to promote prostate cancer cell invasion. *Int J Oncol.* **40**, 1714-1724 (2012).

53. Hu, P. *et al*. Multiplexed quantum dot labeling of activated c-Met signaling in castration-resistant human prostate cancer. *PLoS One.* **6**, e28670 (2011).

54. Ge, D. *et al*. LNCaP prostate cancer cells with autocrine interleukin-6 expression are resistant to IL-6-induced neuroendocrine differentiation due to increased expression of suppressors of cytokine signaling. *Prostate .* **72**, 1306-1316 (2012).

55. Miles, F.L., Tung, N.S., Aguiar, A.A., Kurtoglu, S. & Sikes RA. Increased TGF-β1-mediated suppression of growth and motility in castrate-resistant prostate cancer cells is consistent with Smad2/3 signaling. *Prostate.* **72**, 1339-1350 (2012).

56. Yamamura, S. *et al*. MicroRNA-34a modulates c-Myc transcriptional complexes to suppress malignancy in human prostate cancer cells. *PLoS One.* **7**: e29722 (2012).

57. Lessard, L. *et al*. PTP1B is an androgen receptor-regulated phosphatase that promotes the progression of prostate cancer. *Cancer Res.* **72**, 1529-1537 (2012).

58. Takayama, K. *et al*. TACC2 is an androgen-responsive cell cycle regulator promoting androgen-mediated and castration-resistant growth of prostate cancer. *Mol Endocrinol.* **26**, 748-761 (2012).

59. Yang, P. *et al*. Histone methyltransferase NSD2/MMSET mediates constitutive NF-κB signaling for cancer cell proliferation, survival, and tumor growth via a feed-forward loop. *Mol Cell Biol.* **32**, 3121-3131 (2012).

60. Sen, A. *et al*. Paxillin mediates extranuclear and intranuclear signaling in prostate cancer proliferation. *J Clin Invest .* **122**, 2469-2481 (2012).

61. Wang, J. *et al*. B-Raf activation cooperates with PTEN loss to drive c-Myc expression in advanced prostate cancer. *Cancer Res.* **72**, 4765-4776 (2012).

62. Sampson, N., Ruiz, C., Zenzmaier, C., Bubendorf, L. & Berger, P. PAGE4 positivity is associated with attenuated AR signaling and predicts patient survival in hormone-naive prostate cancer. *Am J Pathol.* **181**, 1443-1454 (2012).

63. Al-Azayzih, A., Gao, F., Goc, A. & Somanath, P.R. TGFβ1 induces apoptosis in invasive prostate cancer and bladder cancer cells via Akt-independent, p38 MAPK and JNK/SAPK-mediated activation of caspases. *Biochem Biophys Res Commun.*  ***427***, 165-170 (2012).

64. He, H.J. *et al*. Krüppel-like factor 8 is a novel androgen receptor co-activator in human prostate cancer. *Acta Pharmacol Sin.* **34**, 282-288 (2013).

65. Hofland, J. *et al*. Activin A stimulates AKR1C3 expression and growth in human prostate cancer. *Endocrinology.* **153**, 5726-5734 (2012).

66. Kuo, P.L., Shen, K.H., Hung, S.H. & Hsu, Y.L. CXCL1/GROα increases cell migration and invasion of prostate cancer by decreasing fibulin-1 expression through NF-κB/HDAC1 epigenetic regulation. *Carcinogenesis.* **33,** 2477-2487 (2012).

67. Killian, P.H. *et al*. Curcumin inhibits prostate cancer metastasis in vivo by targeting the inflammatory cytokines CXCL1 and -2. *Carcinogenesis.* **33,** 2507-2519 (2012).

68. Wu, F., Peacock, S.O., Rao, S., Lemmon, S.K. & Burnstein, K.L. Novel interaction between the co-chaperone Cdc37 and Rho GTPase exchange factor Vav3 promotes androgen receptor activity and prostate cancer growth. *J Biol Chem.* **288**, 5463-5474 (2013).

69. Wu, L. *et al*. CCN3/NOV gene expression in human prostate cancer is directly suppressed by the androgen receptor. *Oncogene.* **33,** 504-513 (2014).

70. Kregel, S. *et al*. Sox2 is an androgen receptor-repressed gene that promotes castration-resistant prostate cancer. *PLoS One.* **8,** e53701 (2013).

71. Wang, Y., Radhakrishnan, D., He, X., Peehl, D.M. & Eng, C. Transcription factor KLLN inhibits tumor growth by AR suppression, induces apoptosis by TP53/TP73 stimulation in prostate carcinomas, and correlates with cellular differentiation. *J Clin Endocrinol Metab.* **98**, E586-594 (2013).

72. Bohrer, L.R. *et al*. FOXO1 binds to the TAU5 motif and inhibits constitutively active androgen receptor splice variants. *Prostate.* **73**, 1017-1027 (2013).

73. Lu, W., Xie, Y., Ma, Y., Matusik, R.J. & Chen, Z. ARF represses androgen receptor transactivation in prostate cancer. *Mol Endocrinol .* **27**, 635-648 (2013).

74. Vo, B.T. *et al*. TGF-β effects on prostate cancer cell migration and invasion are mediated by PGE2 through activation of PI3K/AKT/mTOR pathway. *Endocrinology.* **154**, 1768-1779 (2013).

75. Wang, X. *et al*. Endothelial cells enhance prostate cancer metastasis via IL-6→androgen receptor→TGF-β→MMP-9 signals. *Mol Cancer Ther.* **12**,1026-37 (2013).

76. Jin, F. *et al*. ERK and AKT signaling drive MED1 overexpression in prostate cancer in association with elevated proliferation and tumorigenicity. *Mol Cancer Res .* **11**, 736-747 (2013).

77. [Wu, K](http://www.ncbi.nlm.nih.gov/pubmed?term=Wu K%5BAuthor%5D&cauthor=true&cauthor_uid=23604126). *et al*. The role of DAB2IP in androgen receptor activation during prostate cancer progression. [*Oncogene*](http://www.ncbi.nlm.nih.gov/pubmed/?term=23604126)*.* **33**, 1954-1963 (2014).

78. [Tien, J.C](http://www.ncbi.nlm.nih.gov/pubmed?term=Tien JC%5BAuthor%5D&cauthor=true&cauthor_uid=23650284). *et al*. The steroid receptor coactivator-3 is required for the development of castration-resistant prostate cancer. [*Cancer Res*](http://www.ncbi.nlm.nih.gov/pubmed/?term=23650284)*.* **73**, 3997-4008 (2013).

79.[Yoo, J.Y](http://www.ncbi.nlm.nih.gov/pubmed?term=Yoo JY%5BAuthor%5D&cauthor=true&cauthor_uid=23669876). *et al*. CK2-NCoR signaling cascade promotes prostate tumorigenesis. [*Oncotarget*](http://www.ncbi.nlm.nih.gov/pubmed/?term=23669876)*.* **4**, 972-983 (2013).

80. [Lee, G.T](http://www.ncbi.nlm.nih.gov/pubmed?term=Lee GT%5BAuthor%5D&cauthor=true&cauthor_uid=23710822). *et al*. Bone morphogenetic protein-6 induces castration resistance in prostate cancer cells through tumor infiltrating macrophages. [*Cancer Sci*](http://www.ncbi.nlm.nih.gov/pubmed/?term=23710822)*.* **104**, 1027-1032 (2013).

81. [Wang, H](http://www.ncbi.nlm.nih.gov/pubmed?term=Wang H%5BAuthor%5D&cauthor=true&cauthor_uid=23769744). *et al*. Stabilization of Snail through AKT/GSK-3β signaling pathway is required for TNF-α-induced epithelial-mesenchymal transition in prostate cancer PC3 cells. [*Eur J Pharmacol*](http://www.ncbi.nlm.nih.gov/pubmed/?term=23769744) *.* **714**, 48-55 (2013).

82. [Ammirante, M](http://www.ncbi.nlm.nih.gov/pubmed?term=Ammirante M%5BAuthor%5D&cauthor=true&cauthor_uid=23796898). *et al*. An IKKα-E2F1-BMI1 cascade activated by infiltrating B cells controls prostate regeneration and tumor recurrence. [*Genes Dev*](http://www.ncbi.nlm.nih.gov/pubmed/?term=23796898)*.* **27**, 1435-1440 (2013).

83. [McGrath, M.J](http://www.ncbi.nlm.nih.gov/pubmed?term=McGrath MJ%5BAuthor%5D&cauthor=true&cauthor_uid=23801747). *et al*. Regulation of the transcriptional coactivator FHL2 licenses activation of the androgen receptor in castrate-resistant prostate cancer. [*Cancer Res*](http://www.ncbi.nlm.nih.gov/pubmed/?term=23801747)*.* **73**, 5066-5079 (2013).

84. [Wu, K](http://www.ncbi.nlm.nih.gov/pubmed?term=Wu K%5BAuthor%5D&cauthor=true&cauthor_uid=23838317). *et al*. The mechanism of DAB2IP in chemoresistance of prostate cancer cells. [*Clin Cancer Res*](http://www.ncbi.nlm.nih.gov/pubmed/?term=23838317)*.* **19**, 4740-4749 (2013).

85. [Manna, S](http://www.ncbi.nlm.nih.gov/pubmed?term=Manna S%5BAuthor%5D&cauthor=true&cauthor_uid=23894194). et al. Proteasome inhibition by bortezomib increases IL-8 expression in androgen-independent prostate cancer cells: the role of IKKα. [*J Immunol*](http://www.ncbi.nlm.nih.gov/pubmed/?term=23894194)*.* **191**, 2837-28346 (2013).

86. [Fredericks, W.J](http://www.ncbi.nlm.nih.gov/pubmed?term=Fredericks WJ%5BAuthor%5D&cauthor=true&cauthor_uid=23919967). *et al*. The tumor suppressor TERE1 (UBIAD1) prenyltransferase regulates the elevated cholesterol phenotype in castration resistant prostate cancer by controlling a program of ligand dependent SXR target genes. [*Oncotarget*](http://www.ncbi.nlm.nih.gov/pubmed/?term=23919967) *.* **4**, 1075-1092 (2013).

87. [Zhu, Y](http://www.ncbi.nlm.nih.gov/pubmed?term=Zhu Y%5BAuthor%5D&cauthor=true&cauthor_uid=23922223). *et al*. RhoGDIα downregulates androgen receptor signaling in prostate cancer cells. [*Prostate*](http://www.ncbi.nlm.nih.gov/pubmed/?term=23922223)*.* **73**, 1614-1622 (2013).

88. [Li, X](http://www.ncbi.nlm.nih.gov/pubmed?term=Li X%5BAuthor%5D&cauthor=true&cauthor_uid=23951060). *et al*. MicroRNA-185 and 342 inhibit tumorigenicity and induce apoptosis through blockade of the SREBP metabolic pathway in prostate cancer cells. [*PLoS One*](http://www.ncbi.nlm.nih.gov/pubmed/?term=23951060)*.* **8**, e70987 (2013).

89. [Iglesias-Gato, D](http://www.ncbi.nlm.nih.gov/pubmed?term=Iglesias-Gato D%5BAuthor%5D&cauthor=true&cauthor_uid=24031028). *et al*. SOCS2 mediates the cross talk between androgen and growth hormone signaling in prostate cancer. [*Carcinogenesis*](http://www.ncbi.nlm.nih.gov/pubmed/?term=24031028)*.* **35**, 24-33 (2014).

90. [Wang, Q](http://www.ncbi.nlm.nih.gov/pubmed?term=Wang Q%5BAuthor%5D&cauthor=true&cauthor_uid=24052624). *et al*. Targeting amino acid transport in metastatic castration-resistant prostate cancer: effects on cell cycle, cell growth, and tumor development. [*J Natl Cancer Inst*](http://www.ncbi.nlm.nih.gov/pubmed/?term=24052624) *.* **105**, 1463-1473 (2013).

91. [Chen, R](http://www.ncbi.nlm.nih.gov/pubmed?term=Chen R%5BAuthor%5D&cauthor=true&cauthor_uid=24054868). *et al*. Cav1.3 channel α1D protein is overexpressed and modulates androgen receptor transactivation in prostate cancers. [*Urol Oncol*](http://www.ncbi.nlm.nih.gov/pubmed/?term=24054868)*.* **32**, 524-536 (2014).

92. [Parrondo, R](http://www.ncbi.nlm.nih.gov/pubmed?term=Parrondo R%5BAuthor%5D&cauthor=true&cauthor_uid=24058878)., [de Las Pozas, A](http://www.ncbi.nlm.nih.gov/pubmed?term=de Las Pozas A%5BAuthor%5D&cauthor=true&cauthor_uid=24058878)., [Reiner, T](http://www.ncbi.nlm.nih.gov/pubmed?term=Reiner T%5BAuthor%5D&cauthor=true&cauthor_uid=24058878). & [Perez-Stable, C](http://www.ncbi.nlm.nih.gov/pubmed?term=Perez-Stable C%5BAuthor%5D&cauthor=true&cauthor_uid=24058878). ABT-737, a small molecule Bcl-2/Bcl-xL antagonist, increases antimitotic-mediated apoptosis in human prostate cancer cells. [*PeerJ*](http://www.ncbi.nlm.nih.gov/pubmed/?term=24058878) *.* **1**, e144 (2013).

93.[Ni, J](http://www.ncbi.nlm.nih.gov/pubmed?term=Ni J%5BAuthor%5D&cauthor=true&cauthor_uid=24076216). *et al*. Epithelial cell adhesion molecule (EpCAM) is associated with prostate cancer metastasis and chemo/radioresistance via the PI3K/Akt/mTOR signaling pathway. [*Int J Biochem Cell Biol*](http://www.ncbi.nlm.nih.gov/pubmed/?term=24076216)*.* **45**, 2736-2748 (2013).

94. [Kim, Y.R](http://www.ncbi.nlm.nih.gov/pubmed?term=Kim YR%5BAuthor%5D&cauthor=true&cauthor_uid=24096478). *et al*. HOXB13 downregulates intracellular zinc and increases NF-κB signaling to promote prostate cancer metastasis. [*Oncogene*](http://www.ncbi.nlm.nih.gov/pubmed/?term=24096478)*.* **33**, 4558-4567 (2014).

95. [Kocić, J](http://www.ncbi.nlm.nih.gov/pubmed?term=Kocić J%5BAuthor%5D&cauthor=true&cauthor_uid=24278749)., [Villar, V](http://www.ncbi.nlm.nih.gov/pubmed?term=Villar V%5BAuthor%5D&cauthor=true&cauthor_uid=24278749)., [Krstić, A](http://www.ncbi.nlm.nih.gov/pubmed?term=Krstić A%5BAuthor%5D&cauthor=true&cauthor_uid=24278749). & [Santibanez, J.F](http://www.ncbi.nlm.nih.gov/pubmed?term=Santibanez JF%5BAuthor%5D&cauthor=true&cauthor_uid=24278749). SKIP Downregulation Increases TGF-β1-Induced Matrix Metalloproteinase-9 Production in Transformed Keratinocytes. [*Scientifica (Cairo)*](http://www.ncbi.nlm.nih.gov/pubmed/?term=24278749)*.* **2012**, 861647 (2012).

96. [Robinson, JL](http://www.ncbi.nlm.nih.gov/pubmed?term=Robinson JL%5BAuthor%5D&cauthor=true&cauthor_uid=24292680). *et al*. Elevated levels of FOXA1 facilitate androgen receptor chromatin binding resulting in a CRPC-like phenotype. [*Oncogene*](http://www.ncbi.nlm.nih.gov/pubmed/?term=24292680)*.* **33**, 5666-5674 (2013).

97. [He, W](http://www.ncbi.nlm.nih.gov/pubmed?term=He W%5BAuthor%5D&cauthor=true&cauthor_uid=24294372). *et al*. KAT5 and KAT6B are in positive regulation on cell proliferation of prostate cancer through PI3K-AKT signaling. [*Int J Clin Exp Pathol*](http://www.ncbi.nlm.nih.gov/pubmed/?term=24294372)*.* **6**, 2864-2871 (2013).

98. [Li, B](http://www.ncbi.nlm.nih.gov/pubmed?term=Li B%5BAuthor%5D&cauthor=true&cauthor_uid=24347472). *et al*. Skp2 regulates androgen receptor through ubiquitin-mediated degradation independent of Akt/mTOR pathways in prostate cancer. [*Prostate*](http://www.ncbi.nlm.nih.gov/pubmed/?term=24347472)*.*  ***74***, 421-432 (2014).

99. [Shaikhibrahim, Z](http://www.ncbi.nlm.nih.gov/pubmed?term=Shaikhibrahim Z%5BAuthor%5D&cauthor=true&cauthor_uid=24374838). *et al*. MED15, encoding a subunit of the mediator complex, is overexpressed at high frequency in castration-resistant prostate cancer. [*Int J Cancer*](http://www.ncbi.nlm.nih.gov/pubmed/?term=24374838)*.* **135**, 19-26 (2014).

100. [Lamoureux, F](http://www.ncbi.nlm.nih.gov/pubmed?term=Lamoureux F%5BAuthor%5D&cauthor=true&cauthor_uid=24411988). *et al*. Suppression of heat shock protein 27 using OGX-427 induces endoplasmic reticulum stress and potentiates heat shock protein 90 inhibitors to delay castrate-resistant prostate cancer. [*Eur Urol*](http://www.ncbi.nlm.nih.gov/pubmed/?term=24411988)*.* **66**, 145-155 (2014).

101. [Kim, Y](http://www.ncbi.nlm.nih.gov/pubmed?term=Kim Y%5BAuthor%5D&cauthor=true&cauthor_uid=24441043)., [Kim, J](http://www.ncbi.nlm.nih.gov/pubmed?term=Kim J%5BAuthor%5D&cauthor=true&cauthor_uid=24441043)., [Jang, S.W](http://www.ncbi.nlm.nih.gov/pubmed?term=Jang SW%5BAuthor%5D&cauthor=true&cauthor_uid=24441043). & [Ko, J](http://www.ncbi.nlm.nih.gov/pubmed?term=Ko J%5BAuthor%5D&cauthor=true&cauthor_uid=24441043). The role of sLZIP in cyclin D3-mediated negative regulation of androgen receptor transactivation and its involvement in prostate cancer. [*Oncogene*](http://www.ncbi.nlm.nih.gov/pubmed/?term=24441043)*.* **34**, 226-236 (2015).

102. [Schultz, M.A](http://www.ncbi.nlm.nih.gov/pubmed?term=Schultz MA%5BAuthor%5D&cauthor=true&cauthor_uid=24466341). *et al*. Nrf1 and Nrf2 transcription factors regulate androgen receptor transactivation in prostate cancer cells. [*PLoS One*](http://www.ncbi.nlm.nih.gov/pubmed/?term=24466341)*.* **9**, e87204 (2014).

103. [Ding, L](http://www.ncbi.nlm.nih.gov/pubmed?term=Ding L%5BAuthor%5D&cauthor=true&cauthor_uid=24491799). *et al*. CBP loss cooperates with PTEN haploinsufficiency to drive prostate cancer: implications for epigenetic therapy. [*Cancer Res*](http://www.ncbi.nlm.nih.gov/pubmed/?term=24491799)*.* **74**, 2050-2061 (2014).

104. [Teishima, J](http://www.ncbi.nlm.nih.gov/pubmed?term=Teishima J%5BAuthor%5D&cauthor=true&cauthor_uid=24511001). *et al*. Accumulation of FGF9 in prostate cancer correlates with epithelial-to-mesenchymal transition and induction of VEGF-A expression. [*Anticancer Res*](http://www.ncbi.nlm.nih.gov/pubmed/?term=24511001)*.* **34**, 695-700 (2014).

105. [Lee, G.T](http://www.ncbi.nlm.nih.gov/pubmed?term=Lee GT%5BAuthor%5D&cauthor=true&cauthor_uid=24518599). *et al*. Prostate cancer bone metastases acquire resistance to androgen deprivation via WNT5A-mediated BMP-6 induction. [*Br J Cancer*](http://www.ncbi.nlm.nih.gov/pubmed/?term=24518599)*.* **10**, 1634-1644 (2014).

106. [Tripathi, V](http://www.ncbi.nlm.nih.gov/pubmed?term=Tripathi V%5BAuthor%5D&cauthor=true&cauthor_uid=24683532)., [Popescu, N.C](http://www.ncbi.nlm.nih.gov/pubmed?term=Popescu NC%5BAuthor%5D&cauthor=true&cauthor_uid=24683532). & [Zimonjic, D.B](http://www.ncbi.nlm.nih.gov/pubmed?term=Zimonjic DB%5BAuthor%5D&cauthor=true&cauthor_uid=24683532). DLC1 suppresses NF-κB activity in prostate cancer cells due to its stabilizing effect on adherens junctions. [*Springerplus*](http://www.ncbi.nlm.nih.gov/pubmed/?term=24683532)*.* **3**, 27 (2014).

107. [Kim, I.J](http://www.ncbi.nlm.nih.gov/pubmed?term=Kim IJ%5BAuthor%5D&cauthor=true&cauthor_uid=24898171)., [Kang, T.W](http://www.ncbi.nlm.nih.gov/pubmed?term=Kang TW%5BAuthor%5D&cauthor=true&cauthor_uid=24898171)., [Jeong, T](http://www.ncbi.nlm.nih.gov/pubmed?term=Jeong T%5BAuthor%5D&cauthor=true&cauthor_uid=24898171)., [Kim, Y.R](http://www.ncbi.nlm.nih.gov/pubmed?term=Kim YR%5BAuthor%5D&cauthor=true&cauthor_uid=24898171). & [Jung. C](http://www.ncbi.nlm.nih.gov/pubmed?term=Jung C%5BAuthor%5D&cauthor=true&cauthor_uid=24898171). HOXB13 regulates the prostate-derived Ets factor: implications for prostate cancer cell invasion. [*Int J Oncol*](http://www.ncbi.nlm.nih.gov/pubmed/?term=24898171)*.* **45**, 869-876 (2014).

108. [Tanaka, T](http://www.ncbi.nlm.nih.gov/pubmed?term=Tanaka T%5BAuthor%5D&cauthor=true&cauthor_uid=16762970). *et al*. Modified and bilateral retroperitoneal lymph node dissection for testicular cancer: peri- and postoperative complications and therapeutic outcome. [*Jpn J Clin Oncol*](http://www.ncbi.nlm.nih.gov/pubmed/?term=16762970)*.* **36**, 381-386 (2006).

109. [Liu, Y](http://www.ncbi.nlm.nih.gov/pubmed?term=Liu Y%5BAuthor%5D&cauthor=true&cauthor_uid=20126983)., [Wu, X](http://www.ncbi.nlm.nih.gov/pubmed?term=Wu X%5BAuthor%5D&cauthor=true&cauthor_uid=20126983)., [Dong, Z](http://www.ncbi.nlm.nih.gov/pubmed?term=Dong Z%5BAuthor%5D&cauthor=true&cauthor_uid=20126983). & [Lu, S](http://www.ncbi.nlm.nih.gov/pubmed?term=Lu S%5BAuthor%5D&cauthor=true&cauthor_uid=20126983). The molecular mechanism of Vav3 oncogene on upregulation of androgen receptor activity in prostate cancer cells. [*Int J Oncol*](http://www.ncbi.nlm.nih.gov/pubmed/?term=20126983)*.* **36**, 623-633 (2010).

110. [Nadiminty, N](http://www.ncbi.nlm.nih.gov/pubmed?term=Nadiminty N%5BAuthor%5D&cauthor=true&cauthor_uid=20388792). *et al*. Aberrant activation of the androgen receptor by NF-kappaB2/p52 in prostate cancer cells. [*Cancer Res*](http://www.ncbi.nlm.nih.gov/pubmed/?term=20388792)*.* **70**, 3309-3319 (2010).

111. [Cui, Y](http://www.ncbi.nlm.nih.gov/pubmed?term=Cui Y%5BAuthor%5D&cauthor=true&cauthor_uid=24659479). *et al*. Upregulation of glucose metabolism by NF-κB2/p52 mediates enzalutamide resistance in castration-resistant prostate cancer cells. [*Endocr Relat Cancer*](http://www.ncbi.nlm.nih.gov/pubmed/?term=24659479)*.* **21**, 435-442 (2014).

112. [Li, J](http://www.ncbi.nlm.nih.gov/pubmed?term=Li J%5BAuthor%5D&cauthor=true&cauthor_uid=15897888)., [Wang, E](http://www.ncbi.nlm.nih.gov/pubmed?term=Wang E%5BAuthor%5D&cauthor=true&cauthor_uid=15897888)., [Rinaldo, F](http://www.ncbi.nlm.nih.gov/pubmed?term=Rinaldo F%5BAuthor%5D&cauthor=true&cauthor_uid=15897888). & [Datta, K](http://www.ncbi.nlm.nih.gov/pubmed?term=Datta K%5BAuthor%5D&cauthor=true&cauthor_uid=15897888). Upregulation of VEGF-C by androgen depletion: the involvement of IGF-IR-FOXO pathway. [*Oncogene*](http://www.ncbi.nlm.nih.gov/pubmed/?term=15897888)*.* **24**, 5510-5520 (2005).

113. [Zhou, H](http://www.ncbi.nlm.nih.gov/pubmed?term=Zhou H%5BAuthor%5D&cauthor=true&cauthor_uid=21965718)., [Zhang Y](http://www.ncbi.nlm.nih.gov/pubmed?term=Zhang Y%5BAuthor%5D&cauthor=true&cauthor_uid=21965718). & [Hamburger, A.W](http://www.ncbi.nlm.nih.gov/pubmed?term=Hamburger AW%5BAuthor%5D&cauthor=true&cauthor_uid=21965718). EBP1 inhibits translation of androgen receptor mRNA in castration resistant prostate cancer cells. [*Anticancer Res*](http://www.ncbi.nlm.nih.gov/pubmed/?term=21965718)*.* **31**, 3129-3135 ( 2011).

114. [Rao, S](http://www.ncbi.nlm.nih.gov/pubmed?term=Rao S%5BAuthor%5D&cauthor=true&cauthor_uid=21765461). *et al*. A novel nuclear role for the Vav3 nucleotide exchange factor in androgen receptor coactivation in prostate cancer. [*Oncogene*](http://www.ncbi.nlm.nih.gov/pubmed/?term=21765461)***.* 31**, 716-727 (2012).

115. [Wu, F](http://www.ncbi.nlm.nih.gov/pubmed?term=Wu F%5BAuthor%5D&cauthor=true&cauthor_uid=23281476)., [Peacock, S.O](http://www.ncbi.nlm.nih.gov/pubmed?term=Peacock SO%5BAuthor%5D&cauthor=true&cauthor_uid=23281476)., [Rao, S](http://www.ncbi.nlm.nih.gov/pubmed?term=Rao S%5BAuthor%5D&cauthor=true&cauthor_uid=23281476)., [Lemmon, S.K](http://www.ncbi.nlm.nih.gov/pubmed?term=Lemmon SK%5BAuthor%5D&cauthor=true&cauthor_uid=23281476). & [Burnstein, K.L](http://www.ncbi.nlm.nih.gov/pubmed?term=Burnstein KL%5BAuthor%5D&cauthor=true&cauthor_uid=23281476). Novel interaction between the co-chaperone Cdc37 and Rho GTPase exchange factor Vav3 promotes androgen receptor activity and prostate cancer growth. [*J Biol Chem*](http://www.ncbi.nlm.nih.gov/pubmed/?term=23281476)*.* **288**, 5463-5474 (2013).

116. [Qi, J](http://www.ncbi.nlm.nih.gov/pubmed?term=Qi J%5BAuthor%5D&cauthor=true&cauthor_uid=23518348). *et al*. The E3 ubiquitin ligase Siah2 contributes to castration-resistant prostate cancer by regulation of androgen receptor transcriptional activity. [*Cancer Cell*](http://www.ncbi.nlm.nih.gov/pubmed/?term=23518348)*.* **23**, 332-346 (2013).

117. [Villar, V](http://www.ncbi.nlm.nih.gov/pubmed?term=Villar V%5BAuthor%5D&cauthor=true&cauthor_uid=23766912)., [Kocic, J](http://www.ncbi.nlm.nih.gov/pubmed?term=Kocic J%5BAuthor%5D&cauthor=true&cauthor_uid=23766912). & [Santibanez, J.F](http://www.ncbi.nlm.nih.gov/pubmed?term=Santibanez JF%5BAuthor%5D&cauthor=true&cauthor_uid=23766912). Skip Regulates TGF- β 1-Induced Extracellular Matrix Degrading Proteases Expression in Human PC-3 Prostate Cancer Cells. [*Prostate Cancer*](http://www.ncbi.nlm.nih.gov/pubmed/?term=23766912)*.* **2013**, 398253 (2013).

118. [Cariaga-Martinez, A.E](http://www.ncbi.nlm.nih.gov/pubmed?term=Cariaga-Martinez AE%5BAuthor%5D&cauthor=true&cauthor_uid=23567263). *et al*. Distinct and specific roles of AKT1 and AKT2 in androgen-sensitive and androgen-independent prostate cancer cells. [*Cell Signal*](http://www.ncbi.nlm.nih.gov/pubmed/?term=23567263)*.* **25**, 1586-1597 (2013).

119. [Ewald, J.A](http://www.ncbi.nlm.nih.gov/pubmed?term=Ewald JA%5BAuthor%5D&cauthor=true&cauthor_uid=22937180). & [Jarrard, D.F](http://www.ncbi.nlm.nih.gov/pubmed?term=Jarrard DF%5BAuthor%5D&cauthor=true&cauthor_uid=22937180). Decreased skp2 expression is necessary but not sufficient for therapy-induced senescence in prostate cancer. [*Transl Oncol*](http://www.ncbi.nlm.nih.gov/pubmed/?term=22937180)*.* **5**, 278-287 (2012).

Table S2.

| Name used in the network | Gene symbol | Gene name/official full name |
| --- | --- | --- |
| 4EBP1 | EIF4EBP1 | eukaryotic translation initiation factor 4E binding protein 1 |
| Ack1 | Ack1 | Acetate kinase |
| ADAM17 | ADAM17 | ADAM metallopeptidase domain 17 |
| AKR1C3 | AKR1C3 | aldo-keto reductase family 1, member C3 |
| AKT | AKT1/2 | v-akt murine thymoma viral oncogene homolog 1/2 |
| AMACR | AMACR | alpha-methylacyl-CoA racemase |
| AR | AR | androgen receptor |
| ASCT1 | SLC1A4 | solute carrier family 1 (glutamate/neutral amino acid transporter), member 4 |
| ASCT2 | SLC1A5 | solute carrier family 1 (neutral amino acid transporter), member 5 |
| AT2R | Agtr2 | angiotensin II receptor, type 2 |
| Bak | BAK1 | BCL2-antagonist/killer 1 |
| Bax | Bax | BCL2-associated X protein |
| BCL2 | BCL2 | B-cell CLL/lymphoma 2 |
| Bcl-xL | BCL2L1 | BCL2-like 1 |
| Bmi1 | Bmi1 | BMI1 proto-oncogene, polycomb ring finger |
| BMP6 | BMP6 | bone morphogenetic protein 6 |
| BTG1 | BTG1 | B-cell translocation gene 1, anti-proliferative |
| CACNA1D | CACNA1D | calcium channel, voltage-dependent, L type, alpha 1D subunit |
| CASP3 | CASP3 | caspase 3, apoptosis-related cysteine peptidase |
| CCNB | CCNB1 | cyclin B1 |
| CCND | CCND1/2 | cyclin D1/2 |
| CCNE | CCNE1 | cyclin E1 |
| Cdc37 | Cdc37 | cell division cycle 37 |
| Cdc42 | Cdc42 | cell division cycle 42 |
| CDK1 | CDK1 | cyclin-dependent kinase 1 |
| cdk2 | cdk2 | cyclin-dependent kinase 2 |
| CDKN1A | CDKN1A | cyclin-dependent kinase inhibitor 1A (p21, Cip1) |
| CDKN1B | CDKN1B | cyclin-dependent kinase inhibitor 1B (p27, Kip1) |
| CK2 | CSNK2A1 | casein kinase 2, alpha 1 polypeptide |
| c-Myc | MYC | v-myc avian myelocytomatosis viral oncogene homolog [Homo sapiens |
| CRAF | RAF1 | Raf-1 proto-oncogene, serine/threonine kinase |
| CXCL1 | CXCL1 | chemokine (C-X-C motif) ligand 1 (melanoma growth stimulating activity, alpha) |
| DAB2IP | DAB2IP | DAB2 interacting protein [Homo sapiens |
| DDIT3 | DDIT3 | DNA-damage-inducible transcript 3 |
| E2F1 | E2F1 | E2F transcription factor 1 |
| EBP1 | PA2G4 | proliferation-associated 2G4, 38kDa |
| E-cadherin | CDH1 | cadherin 1, type 1, E-cadherin (epithelial) |
| ECE1 | ECE1 | endothelin converting enzyme 1 |
| EGF | EGF | epidermal growth factor |
| EGFR | EGFR | epidermal growth factor receptor |
| EGR-1 | EGR1 | early growth response 1 |
| Elk1 | ELK1 | ELK1, member of ETS oncogene family |
| EP300 | EP300 | E1A binding protein p300 |
| EpCAM | EpCAM | epithelial cell adhesion molecule |
| ErbB2 | ErbB2 | v-erb-b2 avian erythroblastic leukemia viral oncogene homolog 2 |
| ErbB3 | ErbB3 | v-erb-b2 avian erythroblastic leukemia viral oncogene homolog 3 |
| ERG | ERG | v-ets avian erythroblastosis virus E26 oncogene homolog |
| ERK | ERK | extracellular regulated MAP kinase |
| EZH2 | EZH2 | enhancer of zeste 2 polycomb repressive complex 2 subunit |
| FGF2 | FGF2 | fibroblast growth factor 2 (basic) |
| FGF9 | FGF9 | fibroblast growth factor 9 |
| FHL2 | FHL2 | four and a half LIM domains 2 |
| Fkbp5 | Fkbp5 | FK506 binding protein 5 |
| FKBP51 | FKBP51 | FK506 binding protein 5 |
| FOXA1 | FOXA1 | forkhead box A1 |
| FoxH1 | FoxH1 | forkhead box H1 |
| FOXO1 | FOXO1 | forkhead box O1 |
| GDIα | ARHGDIA | Rho GDP dissociation inhibitor (GDI) alpha |
| GRP78 | HSPA5 | heat shock 70kDa protein 5 (glucose-regulated protein, 78kDa) |
| HOXB13 | HOXB13 | homeobox B13 |
| Hsp27 | HSPB1 | heat shock 27kDa protein 1 |
| Hsp90 | HSP90AA1 | heat shock protein 90kDa alpha (cytosolic), class A member 1 |
| IGF1 | IGF1 | insulin-like growth factor 1 |
| IKBA | NFKBIA | nuclear factor of kappa light polypeptide gene enhancer in B-cells inhibitor, alpha |
| IKKa | CHUK | conserved helix-loop-helix ubiquitous kinase |
| IKKB | IKBKB | inhibitor of kappa light polypeptide gene enhancer in B-cells, kinase beta |
| IL-4 | IL4 | interleukin 4 |
| IL-6 | IL6 | interleukin 6 |
| IL-8 | CXCL8 | chemokine (C-X-C motif) ligand 8 |
| INPP4B | INPP4B | inositol polyphosphate-4-phosphatase, type II, 105kDa |
| IP-10 | CXCL10 | chemokine (C-X-C motif) ligand 10 |
| JAK2 | JAK2 | Janus kinase 2 |
| JNK | MAPK8 | mitogen-activated protein kinase 8 |
| KAT5 | KAT5 | K(lysine) acetyltransferase 5 |
| KLF8 | KLF8 | Kruppel-like factor 8 |
| KLLN | KLLN | killin, p53-regulated DNA replication inhibitor |
| LAT | LAT | linker for activation of T cells |
| MAPK | MAPK1/2… | mitogen-activated protein kinase… |
| Mcl-1 | MCL1 | myeloid cell leukemia 1 |
| MED1 | MED1 | mediator complex subunit 1 |
| MED15 | MED15 | mediator complex subunit 15 |
| MEK | MAP2K7… | mitogen-activated protein kinase kinase7… |
| MMP-9 | MMP9 | matrix metallopeptidase 9 (gelatinase B, 92kDa gelatinase, 92kDa type IV collagenase) |
| mTOR | MTOR | mechanistic target of rapamycin (serine/threonine kinase) |
| MTUS1 | MTUS1 | microtubule associated tumor suppressor 1 |
| N-cadherin | CDH2 | cadherin 2, type 1, N-cadherin (neuronal) |
| NCOA3 | NCOA3 | nuclear receptor coactivator 3 |
| NCoR | NCOR1/2 | nuclear receptor corepressor 1/2 |
| NFKB | NFKB1/2 | nuclear factor of kappa light polypeptide gene enhancer in B-cells 1/2 |
| Nkx3.1 | Nkx3.1 | NK3 homeobox 1 |
| NOV | NOV | nephroblastoma overexpressed |
| NR1I2 | NR1I2 | nuclear receptor subfamily 1, group I, member 2 |
| Nrf1 | Nrf1 | nuclear respiratory factor 1 |
| Nrf2 | NFE2L2 | nuclear factor, erythroid 2-like 2 |
| PAGE4 | PAGE4 | P antigen family, member 4 (prostate associated) |
| PAR1 | F2R | coagulation factor II (thrombin) receptor |
| PDEF | SPDEF | SAM pointed domain containing ETS transcription factor |
| PGE2 | PTGER2 | prostaglandin E receptor 2 |
| PI3K | PIK3CA/B… | phosphatidylinositol-4,5-bisphosphate 3-kinase, catalytic subunit alpha/ beta… |
| PKA-C | PRKACA | protein kinase, cAMP-dependent, catalytic, alpha |
| PKIB | PKIB | protein kinase (cAMP-dependent, catalytic) inhibitor beta |
| PMA | PMAIP1 | phorbol-12-myristate-13-acetate-induced protein 1 |
| PMEPA1 | PMEPA1 | prostate transmembrane protein, androgen induced 1 |
| PSA | KLK3 | kallikrein-related peptidase 3 |
| PTEN | PTEN | phosphatase and tensin homolog |
| PTK2 | PTK2 | protein tyrosine kinase 2 |
| PTPN1 | PTPN1 | protein tyrosine phosphatase, non-receptor type 1 |
| Rac1 | Rac1 | ras-related C3 botulinum toxin substrate 1 (rho family, small GTP binding protein Rac1) |
| Raf | BRAF | B-Raf proto-oncogene, serine/threonine kinase |
| Ras | KRAS | Kirsten rat sarcoma viral oncogene homolog |
| RGS2 | RGS2 | regulator of G-protein signaling 2 |
| RhoGAP | ARHGAP1 | Rho GTPase activating protein 1 |
| RSK | RPS6KA1 | ribosomal protein S6 kinase, 90kDa, polypeptide 1 |
| S6K | RPS6KB1 | ribosomal protein S6 kinase, 70kDa, polypeptide 1 |
| SIAH2 | SIAH2 | siah E3 ubiquitin protein ligase 2 |
| SIRT1 | SIRT1 | sirtuin 1 |
| SKIP | SNW1 | SNW domain containing 1 |
| Skp2 | Skp2 | S-phase kinase-associated protein 2, E3 ubiquitin protein ligase |
| Smad2 | Smad2 | SMAD family member 2 |
| Smad3 | Smad3 | SMAD family member 3 |
| SMAD4 | SMAD4 | SMAD family member 4 |
| SOCS2 | SOCS2 | suppressor of cytokine signaling 2 |
| SOCS3 | SOCS3 | suppressor of cytokine signaling 3 |
| Sox2 | Sox2 | SRY (sex determining region Y)-box 2 |
| Spry1 | Spry1 | sprouty homolog 1, antagonist of FGF signaling |
| Spry2 | Spry2 | sprouty homolog 2 |
| Src | SRC | SRC proto-oncogene, non-receptor tyrosine kinase |
| SREBP-1 | SREBF1 | sterol regulatory element binding transcription factor 1 |
| Stat3 | STAT3 | signal transducer and activator of transcription 3 (acute-phase response factor) |
| STAT5 | STAT5A | signal transducer and activator of transcription 5A |
| TACC2 | TACC2 | transforming, acidic coiled-coil containing protein 2 |
| TF6 | TFG | TRK-fused gene |
| TCF4 | TCF4 | transcription factor 4 |
| TGFB | TGFB1/2 | transforming growth factor, beta 1/2 |
| TGFBR1 | TGFBR1 | transforming growth factor, beta receptor 1 |
| TMPRSS2 | TMPRSS2 | transmembrane protease, serine 2 |
| TNFa | TNF | tumor necrosis factor |
| TNFSF11 | TNFSF11 | tumor necrosis factor (ligand) superfamily, member 11 |
| TP53 | TP53 | tumor protein p53 |
| TP73 | TP73 | tumor protein p73 |
| TPD52 | TPD52 | tumor protein D52 |
| UBE2C | UBE2C | ubiquitin-conjugating enzyme E2C |
| UBIAD1 | UBIAD1 | UbiA prenyltransferase domain containing 1 |
| Vav3 | VAV3 | vav 3 guanine nucleotide exchange factor |
| VEGFA | VEGFA | vascular endothelial growth factor A |
| WHSC1 | WHSC1 | Wolf-Hirschhorn syndrome candidate 1 |
| Wnt | WNT1/2… | wingless-type MMTV integration site family |
| WNT5A | WNT5A | wingless-type MMTV integration site family, member 5A |
| ZnT4 | SLC30A4 | solute carrier family 30 (zinc transporter), member 4 |

Table S3.

| Name used in the network | Gene symbol | Gene name | deregulation | Ref. |
| --- | --- | --- | --- | --- |
| EGFR | EGFR | epidermal growth factor receptor | Overexpression | 1 |
| Raf | BRAF | B-Raf proto-oncogene, serine/threonine kinase | Elevated levels | 2 |
|  | RAF1 | Raf-1 proto-oncogene, serine/threonine kinase | Elevated levels | 2 |
| MEK | MAP2K7 | mitogen-activated protein kinase kinase 7 | Elevated levels | 2 |
| ERK | MAPK1 | mitogen-activated protein kinase 1 | Elevated levels | 2 |
| ErbB2 | ErbB2 | v-erb-b2 avian erythroblastic leukemia viral oncogene homolog 2 | High expression | 3 |
| TGFB | TGFB1 | transforming growth factor, beta 1 | High expressed | 4 |
|  | TGFB2 | transforming growth factor, beta 2 | High expressed | 4 |
|  | TGFB3 | transforming growth factor, beta 3 | High expressed | 4 |
| FOXH1 | FOXH1 | forkhead box H1 | Lower in PC3 and DU145 | 5 |
| MED1 | MED1 | mediator complex subunit 1 | overexpressed | 6 |
| SIRT1 | SIRT1 | sirtuin 1 | up-regulated | 7 |
| CDKN2A | CDKN2A | cyclin-dependent kinase inhibitor 2A | Tumor suppressor | 8 |
| UBE2C | UBE2C | ubiquitin-conjugating enzyme E2C | overexpressed | 9 |
| PTEN | PTEN | phosphatase and tensin homolog | Loss expression | 10 |
| NKX3.1 | NKX3.1 | NK3 homeobox 1 | Reduced expression | 11 |
| Bmi1 | Bmi1 | BMI1 proto-oncogene, polycomb ring finger | recruitment | 12 |
| IL-6 | IL-6 | interleukin 6 | growth | 13 |
| TNFa | TNF | tumor necrosis factor | Higher expression | 14 |
| CCND | CCND1 | cyclin D1 | overexpression | 15 |
| CACNA1D | CACNA1D | calcium channel, voltage-dependent, L type, alpha 1D subunit | highly expressed | 16 |
| TNFSF11 | TNFSF11 | tumor necrosis factor (ligand) superfamily, member 11 | increased | 17 |
| EBP1 | PA2G4 | proliferation-associated 2G4, 38kDa | significantly reduced | 18 |
| STAT3 | STAT3 | signal transducer and activator of transcription 3 (acute-phase response factor) | active | 19 |
| JAK2 | JAK2 | Janus kinase 2 | expression | 20 |
| CXCL1 | CXCL1 | chemokine (C-X-C motif) ligand 1 (melanoma growth stimulating activity, alpha) | overexpressed | 21 |
| BMP-6 | BMP6 | bone morphogenetic protein 6 | dramatic induction | 22 |
| IKKa | CHUK | conserved helix-loop-helix ubiquitous kinase | activate | 12 |
| c-Myc | MYC | v-myc avian myelocytomatosis viral oncogene homolog | Tumor expression | 23 |
| PI3K | PI3K | phosphatidylinositide 3-kinases | Elevated levels | 2 |
| AKT | AKT1 | v-akt murine thymoma viral oncogene homolog 1 | Elevated levels | 2 |
|  | AKT2 | v-akt murine thymoma viral oncogene homolog 2 | Elevated levels | 2 |
| mTOR | mTOR | mechanistic target of rapamycin (serine/threonine kinase) | Elevated levels | 2 |
| SKP2 | SKP2 | S-phase kinase-associated protein 2, E3 ubiquitin protein ligase | overexpression | 24 |
| Vav3 | Vav3 | vav 3 guanine nucleotide exchange factor | High expression | 25 |
| INPP4B | INPP4B | inositol polyphosphate-4-phosphatase, type II, 105kDa | tumor suppressor | 26 |
| EGR-1 | EGR1 | early growth response 1 | loss | 27 |
| BCL-2 | BCL2 | B-cell CLL/lymphoma 2 | high levels | 28 |
| PDEF | SPDEF | SAM pointed domain containing ETS transcription factor | expression | 29 |
| Bcl-XL | BCL2L1 | BCL2-like 1 | high levels | 30 |
| IL-4 | Il4 | interleukin 4 | significantly elevated | 31 |
| EP300 | EP300 | E1A binding protein p300 | highly expressed | 32 |
| DAB2IP | DAB2IP | DAB2 interacting protein | Loss | 33 |
| WHSC1 | WHSC1 | Wolf-Hirschhorn syndrome candidate 1 | overexpression | 34 |
| E2F1 | E2F1 | E2F transcription factor 1 | active | 12 |
| IGF-1 | IGF-1 | insulin-like growth factor 1 (somatomedin C) | increased | 35 |
| IL-8 | CXCL8 | chemokine (C-X-C motif) ligand 8 | Higher expression | 14 |
| SOCS2 | SOCS2 | suppressor of cytokine signaling 2 | Tumor suppressor | 36 |

**REFERENCE**

1. Patel, B.J. *et al*. CL1-GFP: an androgen independent metastatic tumor model for prostate cancer. *J Urol.* **164**, 1420-1425 (2000).

2. Steelman L.S. *et al*. Roles of the Raf/MEK/ERK and PI3K/PTEN/Akt/mTOR pathways in controlling growth and sensitivity to therapy-implications for cancer and aging. *Aging (Albany NY).* **3**, 192-222 (2011).

3. Shi Y. *et al*. Her-2/neu expression in prostate cancer: high level of expression associated with exposure to hormone therapy and androgen independent disease. *J Uro*. **166**, 1514-1519 (2001).

4. Konrad, L. *et al*. Alternative splicing of TGF-betas and their high-affinity receptors T beta RI, T beta RII and T beta RIII (betaglycan) reveal new variants in human prostatic cells. *BMC Genomics.*  **8**, 318 (2007).

5. Chen, G. *et al*. Modulation of androgen receptor transactivation by FoxH1. A newly identified androgen receptor corepressor. *J Biol Chem.*  **280**, 36355-36363 (2005).

6. Vijayvargia, R., May, M.S. & Fondell, J.D. A coregulatory role for the mediator complex in prostate cancer cell proliferation and gene expression. *Cancer Res.*  **67**, 4034-4041 (2007).

7. Kojima, K., Fujita, Y., Nozawa, Y., Deguchi, T. & Ito, M. MiR-34a attenuates paclitaxel-resistance of hormone-refractory prostate cancer PC3 cells through direct and indirect mechanisms. *Prostate.* **70**, 1501-1512 (2010).

8. Zhang, H. *et al*. FOXO1 inhibits Runx2 transcriptional activity and prostate cancer cell migration and invasion. *Cancer Res.*  **71**, 3257-3267 (2011).

9. Chen, Z. *et al*. Phospho-MED1-enhanced UBE2C locus looping drives castration-resistant prostate cancer growth. *EMBO J*. **30**, 2405-2419 (2011).

10. Ferraldeschi, R. *et al*. PTEN Protein Loss and Clinical Outcome from Castration-resistant Prostate Cancer Treated with Abiraterone Acetate. *Eur Urol.* **14**, 01100-01102 (2014).

11. Rao, V., Guan, B., Mutton, L.N. & Bieberich, C.J. Proline-mediated proteasomal degradation of the prostate-specific tumor suppressor NKX3.1. *J Biol Chem.* **287**, 36331-36340 (2012).

12. Ammirante, M. *et al*. An IKKα-E2F1-BMI1 cascade activated by infiltrating B cells controls prostate regeneration and tumor recurrence. *Genes Dev.* **27**, 1435-1440 (2013).

13. Asbagh, L.A., Uzunoglu, S. & Cal, C. Zoledronic acid effects interleukin-6 expression in hormone-independent prostate cancer cell lines. *Int Braz J Urol*. **34**, 355-363 (2008).

14. Sharma, J. *et al*. Elevated IL-8, TNF-α, and MCP-1 in men with metastatic prostate cancer starting androgen-deprivation therapy (ADT) are associated with shorter time to castration-resistance and overall survival. *Prostate.* **74**, 820-828 (2014).

15. Noel, E.E. *et al*. The association of CCND1 overexpression and cisplatin resistance in testicular germ cell tumors and other cancers. *Am J Pathol.* **176**, 2607-2615 (2010).

16. Chen, R. *et al*. Cav1.3 channel α1D protein is overexpressed and modulates androgen receptor transactivation in prostate cancers. *Urol Oncol.* **32**, 524-536 (2014).

17. Gomez-Veiga, F. *et al*. Advances in prevention and treatment of bone metastases in prostate cancer. Role of RANK/RANKL inhibition. *Actas Urol Esp.* **37**, 292-304 (2013).

18. Zhou, H., Zhang, Y. & Hamburger, A.W. EBP1 inhibits translation of androgen receptor mRNA in castration resistant prostate cancer cells. *Anticancer Res.* **31**, 3129-3135 (2011).

19. Chau, M.N. & Banerjee, P.P. Development of a STAT3 reporter prostate cancer cell line for high throughput screening of STAT3 activators and inhibitors. *Biochem Biophys Res Commun.*  **377**, 627-631 (2008).

20. Gu, L. *et al*. Activating mutation (V617F) in the tyrosine kinase JAK2 is absent in locally-confined or castration-resistant prostate cancer. *Anal Cell Pathol (Amst).* **33**, 55-59 (2010).

21. Kuo, P.L., Shen, K.H., Hung, S.H. & Hsu, Y.L. CXCL1/GROα increases cell migration and invasion of prostate cancer by decreasing fibulin-1 expression through NF-κB/HDAC1 epigenetic regulation. *Carcinogenesis.*  **33**, 2477-2487 (2012).

22. Lee, G.T. *et al*. Prostate cancer bone metastases acquire resistance to androgen deprivation via WNT5A-mediated BMP-6 induction. *Br J Cancer*. **110**, 1634-1644 (2014).

23. Vander Griend, D.J., Litvinov, I.V. & Isaacs, J.T. Conversion of androgen receptor signaling from a growth suppressor in normal prostate epithelial cells to an oncogene in prostate cancer cells involves a gain of function in c-Myc regulation. *Int J Biol Sci.* **10**, 627-642 (2014).

24. Li, B. *et al*. Skp2 regulates androgen receptor through ubiquitin-mediated degradation independent of Akt/mTOR pathways in prostate cancer. *Prostate.* **74**, 421-432 (2014).

25. Peacock, S.O., Fahrenholtz, C.D. & Burnstein, K.L. Vav3 enhances androgen receptor splice variant activity and is critical for castration-resistant prostate cancer growth and survival. *Mol Endocrinol.* **26**, 1967-1979 (2012).

26. Hodgson, M.C. *et al*. Decreased expression and androgen regulation of the tumor suppressor gene INPP4B in prostate cancer. *Cancer Res.* **71**, 572-582 (2011).

27. Wu, K. *et al*. The mechanism of DAB2IP in chemoresistance of prostate cancer cells. *Clin Cancer Res.* **19**, 4740-4749 (2013).

28. Zhang, Y.X. *et al*. Ursolic acid overcomes Bcl-2-mediated resistance to apoptosis in prostate cancer cells involving activation of JNK-induced Bcl-2 phosphorylation and degradation. *J Cell Biochem.* **109**, 764-773 (2010).

29. Kim, I.J., Kang, T.W., Jeong, T., Kim, Y.R. & Jung, C. HOXB13 regulates the prostate-derived Ets factor: implications for prostate cancer cell invasion. *Int J Oncol.* **45**, 869-876 (2014).

30. Lee D.I. *et al*. Mechanisms of resistance and adaptation to thapsigargin in androgen-independent prostate cancer PC3 and DU145 cells. *Arch Biochem Biophys.* **464**, 19-27 (2007).

31. Lee, S.O. *et al*. Interleukin-4 stimulates androgen-independent growth in LNCaP human prostate cancer cells. *Prostate.*  **68**, 85-91 (2008).

32. anter, F.R. *et al*. Inhibition of the acetyltransferases p300 and CBP reveals a targetable function for p300 in the survival and invasion pathways of prostate cancer cell lines. *Mol Cancer Ther.*  **10**, 1644-1655 (2011).

33. Yun, E.J. *et al*. DAB2IP regulates cancer stem cell phenotypes through modulating stem cell factor receptor and ZEB1. *Oncogene.***34**, 2741-2752 (2015)

34. Yang, P. *et al*. Histone methyltransferase NSD2/MMSET mediates constitutive NF-κB signaling for cancer cell proliferation, survival, and tumor growth via a feed-forward loop. *Mol Cell Biol*. **32**, 3121-3131 (2012).

35. Takeuchi, A. et al. Insulin-like growth factor-I induces CLU expression through Twist1 to promote prostate cancer growth. *Mol Cell Endocrinol.*  **384**, 117-125 (2014).

36. Iglesias-Gato, D. *et al*. SOCS2 mediates the cross talk between androgen and growth hormone signaling in prostate cancer. *Carcinogenesis.*  **35**, 24-33 (2014).

Table S4.

| order | Feed-back loops |
| --- | --- |
| 1 | Raf=MEK ERK=MAPK !Akt=Raf MAPK=IL-6 IL-6=NFKB CXCL1=AKT NFKB=CXCL1 MEK=ERK |
| 2 | Raf=MEK ERK=MAPK !Akt=Raf MAPK=IL-6 IL-6=NFKB TGFB=PI3K AR=TGFB MEK=ERK PI3K=AKT NFKB=AR |
| 3 | Raf=MEK ERK=MAPK !Akt=Raf MAPK=IL-6 !Bmi1=CDKN2A CDKN1A=E2F1 NKX3.1=TNFa SMAD3=CDKN1A TGFB=Smad3 IL-6=NFKB !CDKN2A=NKX3.1 AR=TGFB TNFa=AKT E2F1=Bmi1 MEK=ERK NFKB=AR |
| 4 | Raf=MEK ERK=MAPK !Akt=Raf MAPK=IL-6 !Bmi1=CDKN2A CDKN1A=E2F1 NKX3.1=TNFa SMAD4=CDKN1A IL-6=NFKB !CDKN2A=NKX3.1 AR=TGFB TNFa=AKT E2F1=Bmi1 MEK=ERK NFKB=AR TGFB=Smad4 |
| 5 | Raf=MEK ERK=MAPK !Akt=Raf MAPK=IL-6 AR=WHSC1 WHSC1=NFKB CXCL1=AKT NFKB=CXCL1 MEK=ERK IL-6=AR |
| 6 | Raf=MEK ERK=MAPK !Akt=Raf MAPK=IL-6 TGFB=PI3K AR=TGFB MEK=ERK PI3K=AKT IL-6=AR |
| 7 | TGFB=TNFSF11 Raf=MEK ERK=MAPK !Akt=Raf MAPK=IL-6 TNFSF11=NFKB CXCL1=AKT NFKB=CXCL1 AR=TGFB MEK=ERK IL-6=AR |
| 8 | Raf=MEK ERK=MAPK !Akt=Raf MAPK=IL-6 !Bmi1=CDKN2A CDKN1A=E2F1 NKX3.1=TNFa SMAD3=CDKN1A TGFB=Smad3 CXCL1=AKT NFKB=CXCL1 !CDKN2A=NKX3.1 AR=TGFB TNFa=NFKB E2F1=Bmi1 MEK=ERK IL-6=AR |
| 9 | Raf=MEK ERK=MAPK !Akt=Raf MAPK=IL-6 !Bmi1=CDKN2A CDKN1A=E2F1 NKX3.1=TNFa SMAD3=CDKN1A TGFB=Smad3 !CDKN2A=NKX3.1 AR=TGFB TNFa=AKT E2F1=Bmi1 MEK=ERK IL-6=AR |
| 10 | Raf=MEK ERK=MAPK !Akt=Raf MAPK=IL-6 !Bmi1=CDKN2A CDKN1A=E2F1 NKX3.1=TNFa SMAD4=CDKN1A CXCL1=AKT NFKB=CXCL1 !CDKN2A=NKX3.1 AR=TGFB TNFa=NFKB E2F1=Bmi1 MEK=ERK IL-6=AR TGFB=Smad4 |
| 11 | Raf=MEK ERK=MAPK !Akt=Raf MAPK=IL-6 !Bmi1=CDKN2A CDKN1A=E2F1 NKX3.1=TNFa SMAD4=CDKN1A !CDKN2A=NKX3.1 AR=TGFB TNFa=AKT E2F1=Bmi1 MEK=ERK IL-6=AR TGFB=Smad4 |
| 12 | Raf=MEK !Akt=Raf AR=WHSC1 WHSC1=NFKB CXCL1=AKT NFKB=CXCL1 MED1=AR ERK=MED1 MEK=ERK |
| 13 | Raf=MEK !Akt=Raf AR=WHSC1 WHSC1+NFKB=IL-6 IL-6=NFKB CXCL1=AKT NFKB=CXCL1 MED1=AR ERK=MED1 MEK=ERK |
| 14 | Raf=MEK !Akt=Raf TGFB=PI3K AR=TGFB MED1=AR ERK=MED1 MEK=ERK PI3K=AKT |
| 15 | TGFB=TNFSF11 Raf=MEK !Akt=Raf TNFSF11=NFKB CXCL1=AKT NFKB=CXCL1 AR=TGFB MED1=AR ERK=MED1 MEK=ERK |
| 16 | Raf=MEK !Akt=Raf MAPK=IL-6 IL-6=NFKB TGFB=MAPK CXCL1=AKT NFKB=CXCL1 AR=TGFB MED1=AR ERK=MED1 MEK=ERK |
| 17 | Raf=MEK !Akt=Raf !Bmi1=CDKN2A CDKN1A=E2F1 NKX3.1=TNFa SMAD3=CDKN1A TGFB=Smad3 CXCL1=AKT NFKB=CXCL1 !CDKN2A=NKX3.1 AR=TGFB MED1=AR ERK=MED1 TNFa=NFKB E2F1=Bmi1 MEK=ERK |
| 18 | Raf=MEK !Akt=Raf !Bmi1=CDKN2A CDKN1A=E2F1 NKX3.1=TNFa SMAD3=CDKN1A TGFB=Smad3 !CDKN2A=NKX3.1 AR=TGFB MED1=AR ERK=MED1 TNFa=AKT E2F1=Bmi1 MEK=ERK |
| 19 | Raf=MEK !Akt=Raf MAPK=IL-6 !Bmi1=CDKN2A CDKN1A=E2F1 NKX3.1=TNFa SMAD3=CDKN1A TGFB=Smad3 IL-6=NFKB CXCL1=AKT NFKB=CXCL1 !CDKN2A=NKX3.1 AR=TGFB MED1=AR ERK=MED1 TNFa=MAPK E2F1=Bmi1 MEK=ERK |
| 20 | Raf=MEK !Akt=Raf !Bmi1=CDKN2A CDKN1A=E2F1 NKX3.1=TNFa SMAD4=CDKN1A CXCL1=AKT NFKB=CXCL1 !CDKN2A=NKX3.1 AR=TGFB MED1=AR ERK=MED1 TNFa=NFKB E2F1=Bmi1 MEK=ERK TGFB=Smad4 |
| 21 | Raf=MEK !Akt=Raf !Bmi1=CDKN2A CDKN1A=E2F1 NKX3.1=TNFa SMAD4=CDKN1A !CDKN2A=NKX3.1 AR=TGFB MED1=AR ERK=MED1 TNFa=AKT E2F1=Bmi1 MEK=ERK TGFB=Smad4 |
| 22 | Raf=MEK !Akt=Raf MAPK=IL-6 !Bmi1=CDKN2A CDKN1A=E2F1 NKX3.1=TNFa SMAD4=CDKN1A IL-6=NFKB CXCL1=AKT NFKB=CXCL1 !CDKN2A=NKX3.1 AR=TGFB MED1=AR ERK=MED1 TNFa=MAPK E2F1=Bmi1 MEK=ERK TGFB=Smad4 |
| 23 | ERK=MAPK MAPK=IL-6 !Bmi1=CDKN2A NKX3.1=TNFa IL-6=NFKB AKT=IKKA !CDKN2A=NKX3.1 !Fkbp5=AKT AR=Fkbp5 TNFa=ERK E2F1=Bmi1 IKKa=E2F1 NFKB=AR |
| 24 | ERK=MAPK MAPK=IL-6 !Bmi1=CDKN2A NKX3.1=TNFa !INPP4B=AKT AR+NCoR=INPP4B IL-6=NFKB AKT=IKKA !CDKN2A=NKX3.1 TNFa=ERK E2F1=Bmi1 IKKa=E2F1 NFKB=AR |
| 25 | ERK=MAPK MAPK=IL-6 !Bmi1=CDKN2A NKX3.1=TNFa AKT=IKKA !CDKN2A=NKX3.1 !Fkbp5=AKT AR=Fkbp5 TNFa=ERK E2F1=Bmi1 IKKa=E2F1 IL-6=AR |
| 26 | ERK=MAPK MAPK=IL-6 !Bmi1=CDKN2A NKX3.1=TNFa !INPP4B=AKT AR+NCoR=INPP4B AKT=IKKA !CDKN2A=NKX3.1 TNFa=ERK E2F1=Bmi1 IKKa=E2F1 IL-6=AR |
| 27 | Bmi1=CDKN2A NKX3.1=TNFa AKT=IKKA !CDKN2A=NKX3.1 MED1=AR ERK=MED1 !Fkbp5=AKT AR=Fkbp5 TNFa=ERK E2F1=Bmi1 IKKa=E2F1 |
| 28 | !Bmi1=CDKN2A NKX3.1=TNFa !INPP4B=AKT AR+NCoR=INPP4B AKT=IKKA !CDKN2A=NKX3.1 MED1=AR ERK=MED1 TNFa=ERK E2F1=Bmi1 IKKa=E2F1 |
| 29 | AKT=IKKA TGFB=PI3K AR=TGFB BMP-6=IL-6 !IKKa=NFKB NFKB=BMP-6 PI3K=AKT IL-6=AR |
| 30 | AKT=IKKA TGFB=PI3K AR=TGFB !IKKa=NFKB PI3K=AKT NFKB=AR |
| 31 | WHSC1+NFKB=IL-6 AKT=IKKA TGFB=PI3K AR=TGFB !IKKa=NFKB PI3K=AKT IL-6=AR |
| 32 | !Bmi1=CDKN2A CDKN1A=E2F1 NKX3.1=TNFa SMAD3=CDKN1A TGFB=Smad3 AKT=IKKA !CDKN2A=NKX3.1 AR=TGFB BMP-6=IL-6 TNFa=AKT E2F1=Bmi1 !IKKa=NFKB NFKB=BMP-6 IL-6=AR |
| 33 | !Bmi1=CDKN2A CDKN1A=E2F1 NKX3.1=TNFa SMAD3=CDKN1A TGFB=Smad3 AKT=IKKA !CDKN2A=NKX3.1 AR=TGFB TNFa=AKT E2F1=Bmi1 !IKKa=NFKB NFKB=AR |
| 34 | !Bmi1=CDKN2A CDKN1A=E2F1 NKX3.1=TNFa SMAD3=CDKN1A TGFB=Smad3 WHSC1+NFKB=IL-6 AKT=IKKA !CDKN2A=NKX3.1 AR=TGFB TNFa=AKT E2F1=Bmi1 !IKKa=NFKB IL-6=AR |
| 35 | !Bmi1=CDKN2A CDKN1A=E2F1 NKX3.1=TNFa SMAD4=CDKN1A AKT=IKKA !CDKN2A=NKX3.1 AR=TGFB BMP-6=IL-6 TNFa=AKT E2F1=Bmi1 !IKKa=NFKB NFKB=BMP-6 IL-6=AR TGFB=Smad4 |
| 36 | !Bmi1=CDKN2A CDKN1A=E2F1 NKX3.1=TNFa SMAD4=CDKN1A AKT=IKKA !CDKN2A=NKX3.1 AR=TGFB TNFa=AKT E2F1=Bmi1 !IKKa=NFKB NFKB=AR TGFB=Smad4 |
| 37 | !Bmi1=CDKN2A CDKN1A=E2F1 NKX3.1=TNFa SMAD4=CDKN1A WHSC1+NFKB=IL-6 AKT=IKKA !CDKN2A=NKX3.1 AR=TGFB TNFa=AKT E2F1=Bmi1 !IKKa=NFKB IL-6=AR TGFB=Smad4 |
| 38 | MED1=AR AKT=MED1 !Fkbp5=AKT AR=Fkbp5 |
| 39 | !INPP4B=AKT AR+NCoR=INPP4B MED1=AR AKT=MED1 |
| 40 | !Bmi1=CDKN2A AKT=IKKA !CDKN2A=AR !Fkbp5=AKT AR=Fkbp5 E2F1=Bmi1 IKKa=E2F1 |
| 41 | !Bmi1=CDKN2A !INPP4B=AKT AR+NCoR=INPP4B AKT=IKKA !CDKN2A=AR E2F1=Bmi1 IKKa=E2F1 |
| 42 | !Bmi1=CDKN2A NKX3.1=TNFa AKT=IKKA !CDKN2A=NKX3.1 !Fkbp5=AKT AR=Fkbp5 BMP-6=IL-6 TNFa=NFKB E2F1=Bmi1 IKKa=E2F1 NFKB=BMP-6 IL-6=AR |
| 43 | !Bmi1=CDKN2A NKX3.1=TNFa !INPP4B=AKT AR+NCoR=INPP4B AKT=IKKA !CDKN2A=NKX3.1 BMP-6=IL-6 TNFa=NFKB E2F1=Bmi1 IKKa=E2F1 NFKB=BMP-6 IL-6=AR |
| 44 | !Bmi1=CDKN2A NKX3.1=TNFa AKT=IKKA !CDKN2A=NKX3.1 !Fkbp5=AKT AR=Fkbp5 TNFa=NFKB E2F1=Bmi1 IKKa=E2F1 NFKB=AR |
| 45 | !Bmi1=CDKN2A NKX3.1=TNFa !INPP4B=AKT AR+NCoR=INPP4B AKT=IKKA !CDKN2A=NKX3.1 TNFa=NFKB E2F1=Bmi1 IKKa=E2F1 NFKB=AR |
| 46 | !Bmi1=CDKN2A NKX3.1=TNFa WHSC1+NFKB=IL-6 AKT=IKKA !CDKN2A=NKX3.1 !Fkbp5=AKT AR=Fkbp5 TNFa=NFKB E2F1=Bmi1 IKKa=E2F1 IL-6=AR |
| 47 | !Bmi1=CDKN2A NKX3.1=TNFa !INPP4B=AKT AR+NCoR=INPP4B WHSC1+NFKB=IL-6 AKT=IKKA !CDKN2A=NKX3.1 TNFa=NFKB E2F1=Bmi1 IKKa=E2F1 IL-6=AR |
| 48 | MAPK=IL-6 !Bmi1=CDKN2A NKX3.1=TNFa IL-6=NFKB AKT=IKKA !CDKN2A=NKX3.1 !Fkbp5=AKT AR=Fkbp5 TNFa=MAPK E2F1=Bmi1 IKKa=E2F1 NFKB=AR |
| 49 | MAPK=IL-6 !Bmi1=CDKN2A NKX3.1=TNFa !INPP4B=AKT AR+NCoR=INPP4B IL-6=NFKB AKT=IKKA !CDKN2A=NKX3.1 TNFa=MAPK E2F1=Bmi1 IKKa=E2F1 NFKB=AR |
| 50 | MAPK=IL-6 !Bmi1=CDKN2A NKX3.1=TNFa AKT=IKKA !CDKN2A=NKX3.1 !Fkbp5=AKT AR=Fkbp5 TNFa=MAPK E2F1=Bmi1 IKKa=E2F1 IL-6=AR |
| 51 | MAPK=IL-6 !Bmi1=CDKN2A NKX3.1=TNFa !INPP4B=AKT AR+NCoR=INPP4B AKT=IKKA !CDKN2A=NKX3.1 TNFa=MAPK E2F1=Bmi1 IKKa=E2F1 IL-6=AR |
| 52 | !Fkbp5=AKT AR=Fkbp5 BMP-6=IL-6 NFKB=BMP-6 AKT=NFKB IL-6=AR |
| 53 | WHSC1+NFKB=IL-6 !Fkbp5=AKT AR=Fkbp5 AKT=NFKB IL-6=AR |
| 54 | !INPP4B=AKT AR+NCoR=INPP4B BMP-6=IL-6 NFKB=BMP-6 AKT=NFKB IL-6=AR |
| 55 | !INPP4B=AKT AR+NCoR=INPP4B WHSC1+NFKB=IL-6 AKT=NFKB IL-6=AR |
| 56 | !Fkbp5=AKT AR=Fkbp5 NFKB=AR AKT=NFKB |
| 57 | EP300=AR AKT=IKKA !Fkbp5=AKT AR=Fkbp5 E2F1=EP300 IKKa=E2F1 |
| 58 | AKT=AR !Fkbp5=AKT AR=Fkbp5 |
| 59 | !INPP4B=AKT AR+NCoR=INPP4B NFKB=AR AKT=NFKB |
| 60 | EP300=AR !INPP4B=AKT AR+NCoR=INPP4B AKT=IKKA E2F1=EP300 IKKa=E2F1 |
| 61 | AKT=AR !INPP4B=AKT AR+NCoR=INPP4B |
| 62 | CXCL1=AKT AKT=IKKA NFKB=CXCL1 !IKKa=NFKB |

Remark: The node in the left of “=” is the source node, and the node on the right is the target node. “!” denotes the negative regulation.

Table S5.

| order | functions |
| --- | --- |
| 1 | ADAM17 := ADAM17; |
| 2 | EGF := AT2R+1; |
| 3 | EGFR := (ADAM17+1) * (EGF+1)+1; |
| 4 | Ras := EGFR; |
| 5 | Raf := (AKT+1) * ((HSP90+1) * (Ras+1)+1); |
| 6 | MEK := (Raf+1) * (IGF-1+1) * (EGFR+1)+1; |
| 7 | ERK := (PAR1+1) * (TNFa+1) * (TGFB+1) * (MEK+1)+1; |
| 8 | RSK := ERK; |
| 9 | ELK := RSK; |
| 10 | Cdc37 := Cdc37; |
| 11 | HSP90 := Cdc37; |
| 12 | HSP27 := HSP90+1; |
| 13 | ERBB2 := (HSP90+1) * (EGF+1)+1; |
| 14 | ACK1 := HSP90; |
| 15 | MED15 := MED15; |
| 16 | SKIP := SKIP; |
| 17 | TGFB := (AR+1) * (SKIP+1) * (MED15+1)+1; |
| 18 | SMAD4 := TGFB; |
| 19 | SMAD2 := TGFB; |
| 20 | SMAD3 := (TGFB+1) * (MED15+1)+1; |
| 21 | FOXH1 := SMAD2*SMAD4+1; |
| 22 | MED1 := (ERK+1) * (AKT+1)+1; |
| 23 | SIRT1 := FHL2; |
| 24 | FOXO1:= (SIRT1+1)*PTEN; |
| 25 | CDKN2A := Bmi1+1; |
| 26 | UBE2C := MED1; |
| 27 | FHL2 := FHL2; |
| 28 | ERG := ERG; |
| 29 | AT2R := MTUS1; |
| 30 | PTEN := PTEN; |
| 31 | FOXA1 := MED1; |
| 32 | JNK := MAPK; |
| 33 | NKX3.1 := CDKN2A+1; |
| 34 | CASP3 := (NKX3.1*CDKN1B+1) * (JNK+1)+1; |
| 35 | TRAP6 := TRAP6; |
| 36 | PAR1 := TRAP6; |
| 37 | MAPK := (ERK+1) * (Androgen+1)* (EGF+1) * (TGFB+1) * (TNFa+1)+1; |
| 38 | Bmi1 := PTEN * (E2F1+1)+1; |
| 39 | IL-6 := (MAPK+1) * (WHSC1*NFKB +1) * (BMP-6+1)+1 ; |
| 40 | AR := (FOXH1* ERBB2* EGFR* FOXO1* CDKN2A * DAB2IP*EBP1* (SIAH2*NCOR)+1) * ((AKT+1) * (Androgen+1) * (EP300+1) * (Cdc37*Vav3+1) * (MED1+1) * (FHL2+1) * (CACNA1D+1) * (NFKB+1) * (Vav3+1) * (IL-6+1)+1); |
| 41 | TNFa := NKX3.1; |
| 42 | CCND := (AR+1) * (ERBB2+1) * (IL-8+1) * (WHSC1*NFKB +1)+1; |
| 43 | TMPRSS2 := TMPRSS2; |
| 44 | CACNA1D := TMPRSS2*ERG; |
| 45 | TNFSF11 := TGFB; |
| 46 | MTUS1 := MTUS1; |
| 47 | EBP1 := EBP1; |
| 48 | STAT3 := IL-6; |
| 49 | JAK2 := (IL-6+1) * SOCS2+1; |
| 50 | PKIB := PKIB; |
| 51 | PKA-C := PKIB; |
| 52 | CXCL1 := NFKB; |
| 53 | BMP-6 := NFKB; |
| 54 | IKKa := AKT; |
| 55 | AKT := (EBP1 * INPP4B * Fkbp5 +1)* ((HSP90+1) * (PKA-C+1) * * (CXCL1+1) * * (TNFa+1) * (PI3K+1)+1); |
| 56 | c-Myc := AR; |
| 57 | mTOR := AKT; |
| 58 | Androgen := CACNA1D; |
| 59 | MMP-9 := (MAPK+1) * (TGFB+1) * (PDEF+1)+1; |
| 60 | RhoGAP := RhoGAP; |
| 61 | EpCAM := EpCAM; |
| 62 | PI3K := (EGF+1) * (IL-4+1) * PTEN * (TGFB+1) * (EpCAM+1) * (KAT5+1) * (Vav3+1)+1; |
| 63 | Wnt := Wnt; |
| 64 | NFKB := (IKKa+1)* ((EGR-1+1) * (TNFSF11+1) * (WHSC1+1) * (IL-6+1) * (TNFa+1) * (HOXB13+1) * (Wnt+1) * (RhoGAP+1) * (AKT+1) +1); |
| 65 | SKP2 := (c-Myc+1) * (mTOR+1)+1; |
| 66 | PSA := (NFKB+1) * CDKN2A+1; |
| 67 | Vav3 := Vav3; |
| 68 | KAT5 := KAT5; |
| 69 | INPP4B := AR * NCOR; |
| 70 | Fkbp5 := AR; |
| 71 | EGR-1 := IGF-1; |
| 72 | BCL-2 := NFKB； |
| 73 | PDEF := (KAT5+1) * HOXB13+1; |
| 74 | Bcl-XL := (AR+1) * (BCL-2+1)+1; |
| 75 | BAK := Bcl-XL+1; |
| 76 | IL-4 := IL-4; |
| 77 | EP300 := (IL-4+1) * (E2F1+1)+1; |
| 78 | DAB2IP := Wnt; |
| 79 | WHSC1 := AR; |
| 80 | NCOR := CK2+1; |
| 81 | SIAH2 := SIAH2; |
| 82 | CDKN1B := SKP2+1; |
| 83 | ZnT4 := ZnT4; |
| 84 | HOXB13 := ZnT4+1; |
| 85 | E2F1 := (CDKN1A+1) * (IKKa+1)+1; |
| 86 | CDKN1A := (RAC1 * HOXB13+1) *( (SMAD3+1) * (SMAD4+1)+1); |
| 87 | IGF-1 := (DAB2IP+1) * SOCS2+1; |
| 88 | IL-8 := (ERK+1) * (EGR-1+1) * (NFKB+1) +1; |
| 89 | CK2 := CK2; |
| 90 | SOCS2 := Androgen; |
| 91 | RAC1 := RAC1; |
